# Prevalence of anxiety and post-traumatic stress (PTS) among the parents of babies admitted to neonatal units: A systematic review and meta-analysis

**DOI:** 10.1016/j.eclinm.2021.101233

**Published:** 2021-12-21

**Authors:** Reem Malouf, Sian Harrison, Hollie A.L Burton, Chris Gale, Alan Stein, Linda S. Franck, Fiona Alderdice

**Affiliations:** a Policy Research Unit in Maternal and Neonatal Health and Care, National Perinatal Epidemiology Unit, Nuffield Department of Population Health, University of Oxford, Oxford OX3 7LF, UK; b Neonatal Medicine, School of Public Health, Faculty of Medicine, Imperial College London, Chelsea and Westminster campus, 369 Fulham Road, London, SW10 9NH; c Department of Psychiatry, Medical Sciences Division, University of Oxford, Oxford, UK; d Medical Research Council/Wits University Rural Public Health and Health Transitions Research Unit (Agincourt), School of Public Health, Faculty of Health Sciences, University of the Witwatersrand, Johannesburg, South Africa Honorary Professor, African Health Research Institute, KwaZulu, Natal; e Department of Family Health Care Nursing, School of Nursing, University of California San Francisco, 2 Koret Way, San Francisco, CA 94143, USA; f School of Nursing and Midwifery, Queens University Belfast, Belfast, UK

**Keywords:** Neonatal, Parents, Anxiety, PTS, Prevalence

## Abstract

**Background:**

Parents of babies admitted to neonatal units (NNU) are exposed to a range of potentially distressing experiences, which can lead to mental health symptoms such as increased anxiety and post-traumatic stress (PTS). This review aimed to describe how anxiety and PTS are defined and assessed, and to estimate anxiety and PTS prevalence among parents of babies admitted to NNU.

**Method:**

Medline, Embase, PsychoINFO, Cumulative Index to Nursing and Allied Health literature were searched to identify studies published prior to April 14, 2021. Included studies were assessed using Hoy risk of bias tool. A random-effects model was used to estimate pooled prevalence with 95% CIs. Potential sources of variation were investigated using subgroup analyses and meta-regression. The review is registered with PROSPERO (CRD42020162935).

**Findings:**

Fifty six studies involving 6,036 parents met the review criteria; 21 studies assessed anxiety, 35 assessed PTS, and 8 assessed both. The pooled prevalence of anxiety was 41.9% (95%CI:30.9, 53.0) and the pooled prevalence of PTS was 39.9% (95%CI:30.8, 48.9) among parents up to one month after the birth. Anxiety prevalence decreased to 26.3% (95%CI:10.1, 42.5) and PTS prevalence to 24.5% (95%CI:17.4, 31.6) between one month and one year after birth. More than one year after birth PTS prevalence remained high 27.1% (95%CI:20.7, 33.6). Data on anxiety at this time point were limited. There was high heterogeneity between studies and some evidence from subgroup and meta-regression analyses that study characteristics contributed to the variation in prevalence estimates.

**Interpretation:**

The prevalence of anxiety and PTS was high among parents of babies admitted to NNU. The rates declined over time, although they remained higher than population prevalence estimates for women in the perinatal period. Implementing routine screening would enable early diagnosis and effective intervention.

**Funding:**

This research is funded by the National Institute for Health Research (NIHR) Policy Research Programme, conducted through the Policy Research Unit in Maternal and Neonatal Health and Care, PR-PRU-1217-21202. The views expressed are those of the author(s) and not necessarily those of the NIHR or the Department of Health and Social Care.

**Research in Context**

**Evidence before this study**

Survey research has suggested high prevalence of common mental problems among parents whose baby is admitted to a neonatal unit (NNU). No systematic reviews of anxiety and post-traumatic stress (PTS) prevalence in parents admitted to NNU were found following a MEDLINE search.

**Added value of this study**

Fifty-six studies involving 6,036 parents met the review criteria; 21 studies assessed anxiety, 35 assessed PTS, and 8 assessed both. The review findings suggest that anxiety and PTS affect two in five parents of babies admitted to NNU. Both are more prevalent and persistent in parents of babies admitted to NNU in comparison to parents in the general perinatal population. There was high heterogeneity between studies and some evidence that study characteristics contributed to the variation in prevalence estimates.

**Implications of all the available evidence**

The current findings highlight the need for routine mental health screening for parents of babies admitted to NNU as part of standard care in NNU and in the year after birth. Adequate health service resources should be in place to ensure early referral and appropriate interventions are offered. In addition, consideration should be given to making mental health support part of routine care for specific groups, for example, parents of very preterm infants with extended stays.

Alt-text: Unlabelled box

## Introduction

A neonatal unit (NNU) provides integrated services for delivering care to sick and preterm babies in need of specialist care. When a baby is admitted to a NNU the experience can be extremely distressing for the parents. Not only are parents likely to be fearful for their baby's health and survival, they also face separation from their baby, an unfamiliar and possibly overwhelming environment, and potential difficulties accessing information and communicating with staff.^1^^,^^2^ Such experiences may affect parental mental health, which in turn can impact transition to parenthood,^3^, ^4^, ^5^ the parent-infant relationship, and longer-term child development.^6^

Previous research has shown that mental health problems are common among parents of babies admitted to a NNU.^7^, ^8^, ^9^, ^10^ Systematic review evidence estimates the prevalence of depression to be as high as 40% in the early postpartum period among women with premature babies^11^^,^^12^ Less is known about anxiety, as highlighted in a recent scoping review, which found little consistency in prevalence rates across the limited studies reported.^7^ Research on post-traumatic stress (PTS) has increased in the last decade, yet there is still little known about the prevalence of PTS in this population.^7^ There are many challenges for researchers in this field, in particular, the lack of clear, discrete definitions and variability in approaches to measurement of mental health problems in the perinatal period.^13^

There are also complexities in the definition of an NNU and classifications of levels of care differ across countries.^14^^,^^15^ With no internationally agreed definition, synthesising evidence based solely on level of care is problematic. Furthermore, even in comparable NNU settings, parents’ experiences are highly individual and variable, hence it is important to include all parents when exploring parental mental health outcomes. A recent meta-analysis showed that parental stress in NNU is at least partially independent from infants’ risk and suggests that the trauma of being hospitalized in a NNU plays a pivotal role in parents’ stress perception. The review also showed the levels of stress reported by parents only marginally increased as a function of the time spent in the NNU, again highlighting the need for an inclusive approach in research on parental mental health.^16^ Therefore, in this review, the definition of parents of babies admitted to NNU is purposely broad to ensure all parents of infants receiving care in all levels of NNUs are included. Despite the emergence of literature highlighting the potential impact of NNU admission on parents and their babies, there has yet to be a systematic review to estimate prevalence rates of parental anxiety and PTS. Such data are important to inform policy, guide future research, and to ensure clinical practice addresses the mental health needs of parents during and after their baby's NNU admission. This review aimed to fill the gap in the literature by describing how anxiety and PTS are defined and assessed in the research literature and synthesising evidence on the prevalence of anxiety and PTS among parents of babies admitted to NNU.

## Methods

The review was prospectively registered with PROSPERO (CRD42020162935).

### Operational Definitions

NNU: This review includes parents of all babies admitted to NNUs for any level of care.

Anxiety and PTS: Throughout this review, the term ‘anxiety’ is used to describe anxiety symptoms, which can vary from mild to severe^17^ or the presence of an anxiety disorder, such as generalised anxiety disorder. The term ‘PTS’ is used to describe PTS symptoms, which occur in response to an extremely negative or traumatic event, or the presence of acute stress disorder (from three days to one month after the event) or post-traumatic stress disorder (PTSD) (more than one month after the event).

### Search strategy and selection criteria

A search strategy was developed using a combination of free-text (title/abstract) keywords and MeSH (subject terms) to describe the key concepts of anxiety and PTS, parents, NNUs and prevalence. Medline, Embase, PsychoINFO, Cumulative Index to Nursing and Allied Health literature, Web of Science, ResearchGate and Google Scholar were searched (Appendix A). No date or language restrictions were applied. A search of grey literature was conducted using British Library EThOS, Open Grey and ProQuest Dissertations & Theses Global and studies. The websites of not-for-profit organisations Bliss and March of Dimes were also searched for relevant studies. In addition, the reference lists of all included studies were used to identify further relevant publications. The final search was conducted on April 14^th^ 2021.

Studies were included if they: 1) were cohort (prospective or retrospective) or cross-sectional in design; 2) assessed prevalence of anxiety and/or PTS at any time after birth; 3) included mothers, fathers, parents or other primary carers of babies admitted to a NNU.

### Screening, data extraction and risk of bias assessment

Two of three reviewers (RM, SH, FA) independently screened the titles and abstracts of all studies identified by the search. Full texts were independently screened by two of four reviewers applying the review eligibility criteria (RM, SH, HB, FA). Screening was performed using Covidence software.^18^ Disagreements regarding study eligibility were resolved through discussion and consensus within the review team. Study authors were contacted if cut-off points were not reported, if anxiety and/or PTS data were reported as mean scores or combined with prevalence of depression, or if the study was available as an abstract only. Additional information was provided by 14 study authors. Where no data were obtained from authors, missing data were recorded as not reported and subsequently excluded from meta-analyses and meta-regressions.

The following data were extracted for each included study: year and country of publication, study objective, study design, study period, NNU level, infant length of NNU stay, study inclusion/exclusion criteria, demographics of parents and babies, assessment tool, cut-off point, time of assessment and prevalence.

Risk of bias was assessed using the Hoy risk of bias tool (Appendix B).^19^ The tool consists of ten items: items one to four assess external validity (selection bias (items 1-3) and non-response bias (item 4), items five to ten assess internal validity (measurement bias (items 5-9)) and analysis bias (item 10). Appraisal of each item provides a subjective assessment of risk of bias as low, high or unclear. All data were extracted and independently cross-checked by at least two authors (RM, SH, HB, FA).

### Data analysis

Pooled prevalence estimates of anxiety and PTS were calculated by combining estimates from each study. Meta-analysis was conducted using the “Metaprop” function in STATA 15.9.^20^ A random-effects model was applied and the results were reported as proportions with 95% confidence intervals (CI). The data were analysed by time of assessment: up to one month after birth; from one month to one year after birth; and more than one year after birth. If studies reported more than one assessment of anxiety/PTS in the time period, the time point with most participants was included.

Subgroup analyses were pre-specified and conducted across the following study characteristics: setting (high vs. middle income countries), design (cohort vs. cross-sectional), sample representativeness (low vs. high risk of selection bias on item one of the quality assessment vs. no on item one), anxiety measurement tool (self-report vs. clinical interview), sex of parents (male vs. female), self-report scale (State Trait Anxiety Inventory (STAI) state scale vs. others for anxiety; Perinatal Post-Traumatic Stress Disorder Questionnaire (PPQ) vs. others for PTS) and prematurity level (< 33 vs. ≥ 33 weeks gestation). Planned subgroup analyses by birthweight, neonates that had surgical procedures, level of NNU, and length of stay were not feasible due to insufficient data. Sensitivity analysis based on study quality was also planned but could not be performed because no study was low risk of bias on all items.

Evidence of variation in anxiety/PTS prevalence due to between-study heterogeneity was assessed using the I^2^ statistic, which describes the percentage of variation not due to sampling error. An I^2^ value above 50% indicates moderate heterogeneity and above 75% indicates high heterogeneity.^21^ Where there was evidence of high heterogeneity and there were sufficient numbers of studies, meta-regression was performed to investigate whether any variation in prevalence estimates was explained by study characteristics.

**Patient and public involvement (PPI):** A voluntary group of parents, whose babies received care in a neonatal unit and a representative from Bliss provided the PPI input for this review. Parents provided a personal insight from their experience of having a baby cared for in a NNU and worked collaboratively with the team to improve the readability of the scientific language used in the review and interpreting the findings to a wider dissemination of the findings.

### Role of the funding source

The funder of the study had no role in study design, data collection, data analysis, data interpretation, or writing of the report. The corresponding author had full access to all the data in the study and had final responsibility for the decision to submit for publication.

## Results

**Figure 1:**
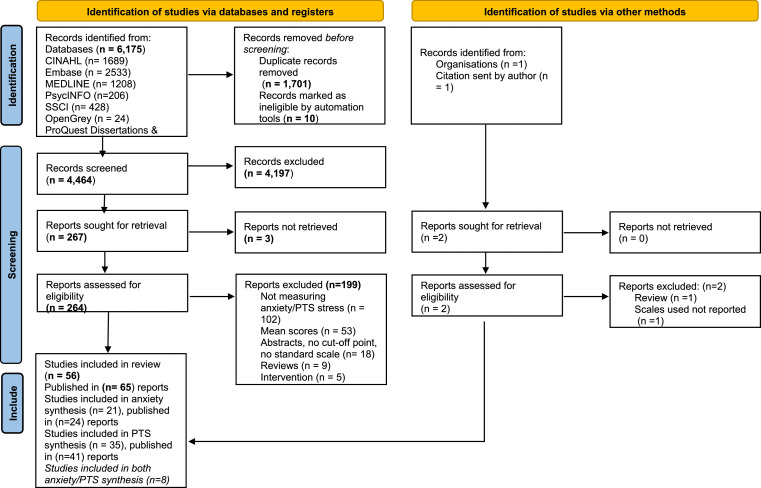
PRISMA flow diagram for study selection *From:* Page MJ, McKenzie JE, Bossuyt PM, Boutron I, Hoffmann TC, Mulrow CD, et al. The PRISMA 2020 statement: an updated guideline for reporting systematic reviews. BMJ 2021;372:n71. doi: 10.1136/bmj.n71. For more information, visit: http://www.prisma-statement.org/

In total, 6,175 relevant records were identified via databases. After the removal of duplicates, the titles and abstracts of 4,464 records were screened and 264 full-text records were assessed against the review eligibility criteria. A total of 56 studies, published in 65 records, met the inclusion criteria. The PRISMA flowchart is presented in Figure 1.

### Anxiety

A description of the included studies is provided in Table 1. In total, 21 studies, published in 24 records,^22^, ^23^, ^24^, ^25^, ^26^, ^27^, ^28^, ^29^, ^30^, ^31^, ^32^, ^33^, ^34^, ^35^, ^36^, ^37^, ^38^, ^39^, ^40^, ^41^, ^42^, ^43^, ^44^, ^45^ reported prevalence of anxiety among 3,639 parents. The sample sizes varied from 29 to 600 participants. Five studies enrolled both mothers and fathers;^24^^,^^33^^,^^35^^,^^40^^,^^43^ 15 studies, published in 18 records, included mothers only;^22^^,^^25^, ^26^, ^27^, ^28^, ^29^, ^30^, ^31^, ^32^^,^^34^^,^^36^, ^37^, ^38^, ^39^^,^^41^^,^^42^^,^^44^^,^^45^ One study included only fathers.*Study characteristics*

**Table 1:** Characteristics of included studies - Anxiety.

**Study ID, country**	**Study design, setting, study period, type of neonatal care, length of stay**	**Study objective**	**Study inclusion criteria**	**Study exclusion criteria**	**Parents’ characteristics**	**Babies’ characteristics**
**Bonacquisti 2020, USA**	Prospective cohort, 3 centres, October 2014 through May 2016, NNU level and length of stay = NR	To identify maternal psychological responses to NNU admission	Mothers ≥18 yrs, given birth less than a year ago to infants admitted to NNUs	Fathers were excluded	N = 127 mothers age = mean 29.63 yrs; living with partner = 69 (54.3%), white = 66 (52%), black = 38 (30%), nulliparous = 52 (40.9%), education (university degree and above) = 26 (20.5%), SES (unemployed) = 40 (31.5%)	N = 147, GA & BW = NR
**Cajiao-Nieto 2021, Spain**	Cohort study, 1 University Hospital in Barcelon, January 2016 to April 2017, NNU= level = NR, length of stay ≥15 days	To compare paternal anxiety symptoms & depression of fathers of PT babies in NNU to fathers of full term babies	Fathers could read & write in Spanish Fathers to babies in NNU for at least 2 weeks	Babies who died or transferred to another hospital	N = 51 fathers, age range 20 -> 45 yrs, all lived with partners, education (professional & superior) = 39 (76%), first-time fathers = 36 (70.2%); SES (employed) = 49 (96%), ethnicity = NR	N= NR, single birth N = 29, GA < 37 wks, BW = NR
**Carter 2007, New Zealand**	Prospective cohort study, 1 central: New Zealand province, Christchurch Women's Hospital, February 2001 to January 2002, NNU level III, length of stay = NR	To compare the psychosocial functioning of the parents of infants admitted to NNU with the parents of infants born at term and not admitted to the NNU	Parents of babies admitted to NNU, criteria for NNU: BW <1800 g, GA <34 wks, or any infant illness	Lack of written informed consent or knowledge that the infant would go into foster care or be adopted	N = 242 mothers, N = 205 fathers. mothers’ age = 30.1 (SD, 5.4), vs. fathers’ age 33.1 (SD, 5.9) yrs, mothers living with partner = 140 (58%), mothers’ professional qualification = 64 (52%) vs fathers: professional qualification = 50 (37%); SES = annual family income(NZ $) < $15000: 16 (7%); parity & ethnicity = NR	N = 276, mean GA = mean 35.1 (SD, 3.8) wks, range = 23-42 wks, BW = 2477 (SD, 889.1)g
**Dantas 2012, Brazil**	Cross-sectional, 2 hospitals, Januário Cicco Maternity School and José Pedro Bezerra and MEJC, located in the municipality of Natal, in Rio Grande do Norte (RN), April to May 2011, NNU &length of stay = NR	To identify the prevalence of symptoms of anxiety and depression in mothers of hospitalized premature infants	Mothers of preterm infants <37wks, admitted to NNU >24 hrs, age ≥18 yrs	Mothers to newborns who died, or with congenital anomaly, drug user, HIV+, mental health illness	N =70 mothers, mean age = 26.50 (range18 – 42) yrs; education: 11 (3-17) yrs; living with partner= 55 (78.6%); SES = (one salary) 17 (24.3%); occupation = 33 (47.1%); parity, SES & ethnicity = NR	N = NR, GA = mean 31.55 wks, range 26- 37 wks, BW = mean 1494g
**Das 2021, USA**	Cross-sectional, 1 hospital in Midwest, study period = NR, NNU level = NR, length of stay = 14-69 days	To determine whether the history of a previously diagnosed	Mothers to babies in NNU for 7–29 days	NR	N = 96 mothers, age range=22-33 yrs, white = 35 (36%), black= 49 (51%), SES = (government insurance) = 84 (88%); living with partner, education & parity = NR	N = 99, BW= 1,285- 3,112 g, GA range = 29-39 wks
**Eutrope 2014**^a^**, France**	Prospective cohort study, 3 hospitals-Reims, Nancy & Besancon, January 2008 to January 2010, 3B NNU (mechanical ventilation, no major surgery) or 3C NNU (major neonatal surgery, no open-heart surgery), length of stay = NR	To describe maternal feelings of delivering a premature baby	Preterm babies admitted to one of the 3 NNU, GA <32 wks	Mothers with psychiatric illness, drug or alcohol abuse, aged <18 years, language barriers; for newborns: unfavourable prognosis PRI≥ 10, significant developmental disabilities malformation and/or genetic anomaly, 30% excluded based on location	N = 100 mothers, mean age = 29.8 (SD,6.0) yrs, 92% lived with partners, higher education 79.29%, SES (employed) = 69%, nulliparous = 48%; ethnicity = NR	N = 100, multiple births = 22 twins & 4 triples; GA = mean 29.8 (SD, 6.0) wks; BW = mean 1320g
**Garfield 2015**^a^**, USA**	Cross-sectional, 2 hospitals inner-city medical centres serving underserved and uninsured populations, study period, NNU level & length of stay = NR	Identifying risk factors among urban, low-income mothers may enable NNU healthcare providers to more effectively screen and refer mothers with potentially elevated postpartum depressive symptom	Mothers of very low BW <1500g, preterm <37, English speaking whose infants were clinically stable	Mothers with mental health diagnosis, babies with congenital neurological problems or symptoms of substance abuse, age <18 yrs, ongoing critical illness = HIV seizure, or diagnosis of major depression, psychosis, bipolar disease. mothers of infants receiving mechanical ventilation	N = 113 mothers, mean age = 24.7 (SD, 5.17), ethnicity African- American = 81%, living with partner = 52.3%, high school graduates = 43%, SES = 39% received public aid and 40% were uninsured, parity = NR	N = NR, GA < 37 wks, BW = mean 1073 (SD, 342)g
**Gonzalez-Hernandez 2019, Mexico**	Cohort study, 1 centre General Hospital of Durango, May 2016 to November 2017, NICU level I, length of stay = ≥1 month	To determine the frequency of depression and anxiety in mothers to NNU babies; to provide socio-demographic characteristics of participants and variables associated with depression and anxiety	Mothers ≥15 yrs, with premature babies in NNU level I, stayed in NNU ≥1month, signed an informed consent	Mothers with a history of previous psychiatric diseases, severe medical illness, babies in NNU levels II, III or IV	N=188 mothers, mean age 24.7 years (S.D, 6.4, range 15–42) yrs, living with partner = 158 (84.0%), bachelor's degree = 9 (4.8%), SES (living in urban areas) = 103 (54.3%), parity & ethnicity = NR	N, GA & BW = NR
**Greene 2015 & 2018**^a^**, USA**	Prospective cohort study,1 NNU, August 2011 to December 2012, NNU-level IV, length of stay = 91 ± 37.1 [30–179] day	To examine multiple types of distress predictors of maternal NNU visitation rates and the relationships between maternal NNU visitation rates and later maternal distress and infant clinic attendance	English-speaking mothers age > 18 years, infants likely to survive assessed by the neonatologist	Congenital anomalies, drug users	N = 69 mothers, age = 26.99 (SD, 5.98) yrs, nulliparous 23 (34%), ethnicity: black = 38 (54%), non-Hispanic white = 18 (26%), Hispanic-white = 12 (17%), Asian 1 (1%); living with partners = 20 (32%), high school education = 32 (48%); SES (Public health insurance) = 44 (66%)	N = 69, GA = mean 27.5 (SD, 2.2) wks (range 23.2-32.30) wks, BW = 957 (SD, 243)g
**Harris 2018**^a^**^,^ USA**	Cohort study, 2 centres Saint Louis Children's Hospital & Barnes Jewish Hospital's Special Care Nursery, January to June 2015, NNU Level III & IV length of stay = 83.4 (40.9) days	To examine the early mental health challenges in mothers of very preterm infants vs mothers of full-terms, identify family social background & infant medical factors associated with high levels of maternal psychological distress & assess the relationship between maternal psychological distress and maternal role	Mothers to infants born ≤32 weeks, free from congenital anomalies	Drug use and younger age were an exclusion criterion	N =37 mothers, age = 29.7 (SD, 6.4) yrs, multiple birth 5 (13%), SES (Low income <$25,000) = 16 (43%), college degree = 15 (41%), single 7 (19%), ethnicity = NR	N = 50, GA ≤32 wks, BW = mean 1104.0 (SD, 416.7)g
**Helle 2016**^a^**^,^ Germany**	Cross-sectional, 3 largest centres of Perinatal Medical Care in Hamburg, (Altona, Barmbek, Eppendorf), study period, NNU level & length of stay = NR	investigating the prevalence of and risk for postpartum anxiety disorders, adjustment disorders and state anxiety four to six weeks postpartum in both parents with a VLBW infant compared to term infants	Mothers to very low birth infants, BW <1500g, GA<37 wks	Inability to follow study procedures, insufficient German language skills, premature discharge, residing too far from the study centre	N = 111 mothers, N = 87 fathers, mothers age = 32.6 (SD 4.66) yrs, fathers age = 23.8 (SD 7.58) yrs, nulliparous= 82 (73.9%), ethnicity = NR, living with partners = 74 (66.7%), SES (low) = 11 (10%)	N =149, GA = mean 28.2 (SD, 2.65) wks, BW = 1095.9 (SD, 330.40)g, singleton = 76 (68.5%), twin = 32 (28.8), triplet = 3 (2.7%)
**Holditch-Davis 2015**^a^**, USA**	Cohort design, NNUs of 4 hospitals (2 in a South-eastern state and 2 in a Midwestern state), NNU level & period = NR	To estimate the inter-correlations between depressive symptoms, state anxiety, PTS, stress due to infant appearance and behaviour, and stress due to parental role alteration in a multi-ethnic sample of m others of preterm infants during initial hospitalization	Mothers of PT babies of BW <1750g	Parents to infants, with congenital neurological anomalies, substance exposure, age< 15yrs; HIV+; psychosis/bipolar disease; depression, critical illness; non-English speaking, follow-up for 12 months was unlikely	N = 232 mothers, Age mean =27.0 yrs (SD 6.1); living with partner = 32.3%; mean education = 13.4 yrs (SD2.3); ethnicity = White = 8% Black = 69.8%, Hispanic = 8,1%, Other =1.9%. Nulliparous = 55.1%.; SES (Public assistance) =20.3%.	N = NR, mean GA=27.2 wks (SD, 2.9), mean BW = 1006.2 (SD, 326)g
**Kong 2013, China**	Cross sectional, 1 centre Department of Paediatrics in Nanjing Maternal and Child Health Hospital, January to September 2011, neonatal care = paediatric department, length of stay> 24 hrs	To investigate parents’ mental health of hospitalised neonates and their characteristics, to measure the stress levels and social support	Parental age ≥18 years, ability to read and write, neonates stayed in hospital >24 hours	Serious physical or mental condition	N = 600 parents, N = 200 mothers, N = 400 fathers, mothers age mean 28.53 ± 4.06 vs. fathers 30.76 ± 4.60 yrs; living with partners years = mothers 3.30 ± 3.13 vs. fathers 3.17 ±2.78 yrs; education = mothers 64% college or higher vs. fathers 73.25%; SES = mothers (low <5000 Yuan per month) 84%, vs. fathers 67.25%, ethnicity & parity = NR	N = 600, GA mean = mothers = 36.63 ± 3.34 vs fathers 37.09 ± 3.16 wks, BW mean: mothers = 2926.70 ± 937.75 vs. fathers = 3051.90 ± 1028.88g
**Misund 2014 & 2016**^a^**, Norway**	Prospective cohort, 1 centre at Oslo University Hospital, Norway; two periods = June 2005–January 2006 and October 2007–July 2008, NNU level & length of stay = NR	To explore the associations between maternal mental health problems following preterm birth, pregnancy and birth complications and early preterm mother–infant interaction at 6 months corrected age	Mothers of preterm babies GA <33 wks admitted to NNU	Mothers of severely ill babies that the medical staff estimated to have poor chance of survival, and non-Norwegian speakers	N = 29 mothers, at first assessment (2 wks after discharge from hospital), N=27 at second assessment (6 months corrected age) & N=26 at third assessment (18 months corrected age), mean age = 33.7 (SD, 4.3) yrs, nulliparous 18 (62.1%), education 12 years = 26 (89.7%), all living with partners, ethnicity &SES = NR	N = 35, GA median (range) = 29 (24-32)wks, mean 28.5 (SD, 2.6), BW median (range)= 1185 (623–2030) mean 1222 (SD, 423)g, multiples = twins 14 (40%) (two sets of twins were raised as singletons due to twin sibling still birth)
**Mulder 2014, New Zealand**	Cohort design,1 centre Christchurch Women's Hospital, NNU serving a region in central Canterbu, February 2001 to January 2002, NNU level & length of stay = NR	To evaluate the psychological functioning in parents whose infants were admitted to a NNU over the first 2 years of the infant's life	NNU admissions born to parents resident in a defined geographic area in a 12-month period were eligible for the study. Criteria for NNU admission BW <1800 g and GA<34 weeks or any illness in the infant	NR	N = 242 families, mothers N=242, mean age = 30.1(SD, 5.4), 88% living with partners, 52% professional qualification. Fathers N=205, mean age=33.1(SD,5.9), 37% professional qualification, parity = NR	GA = 23–42 wks, mean = 35.1 (SD, 3.8) wks, BW = 370–4850g, mean 2477 (SD, 889.1)g
**Ong 2019, Malaysia**	Prospective cohort,1 hospital, study duration 3 years, dates = NR, NNU Level III, mean length of stay = 90.5 (28.6) days	To investigate the demographics, maternal psychosocial and infant factors of mothers of very preterm infants at risk for postpartum depression/anxiety at the time of discharge from a level III (NNU)	Mothers of preterm infants born between 27 to 34 GA wks	Congenital anomaly or being moribund with severe sepsis or respiratory failure in the first days of life	N =73 mothers, mean age 27.2 (SD,7.4)yrs, high school or less =4 6.5%, living with partner = 42.5%, SES (public insurance) = 69%, nulliparous 34.3%, SES = NR	N =73 infants, mean GA =26 wks (SD, 1.8), 27-34 wks BW = NR
**Onay 2021, Turkey**	Cross-sectional, 1 hospital, Eskisehir Osmangazi University Hospital, November 1, 2018 and February 1, 2020, NNU – level III; length of stay ≥7 days	To investigate the relation between breastfeeding exclusivity of NNU infants and the severity of anxiety and depressive symptoms of NNU mothers in early postpartum period	Mothers to preterm and term infants admitted to the NNU	Mothers < 18 years, cannot give breast milk not speaking Turkish, for babies with congenital/ chromosomal abnormalities, inherited metabolic diseases, <7 days NNU, babies who died	N = 93 mothers, mean age = 30.61 yrs, living with partner = 91 (97.8%), education (university)= 26 (28.0%), SES (unemployed) = 68 (73.1), nulliparous = 38 (40.9%), ethnicity = NR	N = 105, GA ≤32 wks = 28 (26.7%), 32–36 wks = 47 (44.8%), > 37 wks = 30 (28.5), BW ≤2,500g = 64 (61%), >2,500g = 41 (39%)
**Pace 2016**^a^**, Australia**	Prospective cohort, 1 centre at Royal Women's Hospital, Melbourne, January 2011 to December 2013, NNU level & Length of stay = NR	To describe the trajectory and predictors of distress in parents of very preterm infants during the first 12 weeks after birth, to compare rates of depression and anxiety in parents of very preterm to term	Families with very preterm infants, GA <30 wks admitted to NNU	Parents who did not speak English, infants with congenital abnormalities, unlikely to survive according to the attending medical team	N= 113 mothers and 101 fathers, mothers age: mean (SD) = 32.7 (5.3) yrs vs. fathers 34.7 (SD, 6.4) yrs, higher social risk all sample= 43%, ethnicity& relationship = NR	N =150 (31 twins, 1 set triplets, 6 died) GA = mean 27.7 (SD, 1.5)wks, BW = mean = 1021(SD, 261)g, Singleton birth = 84 (56%)
**Rogers 2013, USA**	Prospective cohort,1 hospital, 3 year-period, level III urban NNU, length of stay = mean 90.5 (28.6) days	To assess factors for identifying mothers at-risk for postpartum depression or anxiety at the time of NNU discharge among Caucasian and African-American mothers	Mothers to preterm infants born <30 wks	Mothers to babies with congenital anomaly or being moribund with severe sepsis or respiratory failure in the first days of life	N = 73 mothers, age = 27.2 (SD, 7.4) yrs, nulliparous = 34.3%, living with partner= 42.5%, high school or less = 46.5%, SES (public insurance) = 69% Caucasian vs. African American= N=36 vs 37, age 29.5 (SD, .82) vs.25 (5.9) yrs, nulliparous = 34% vs.34.3, living with partner= 73.5% vs. 10.88%,high school or less =37.1 % vs. 55.6%, public insurance 50% vs. 86.5%	N= 73 infants, GA = mean 25.5 (1.8) wks, BW = NR
**Segre 2014 & McCabe-Beane 2018, USA**	Cross-sectional, 1 centre Midwestern academic medical center; December 2010 to May 2012, Level IV NNU, length of stay = NR	To determine whether a diagnostic classification approach or a common-factor model better explained the pattern of symptoms reported by NNU mothers and risk factors of aversive emotional states in NNU mothers based on the supported conceptual model and to expand depression screening to include anxiety symptoms	Mothers to NNU babies, >18 years of age, and English speaking	NR	N = 200 mothers, mean age = 28.1 (SD, 5.7) yrs, ethnicity = Caucasian = 178 (89.9%), African American = 12 (6.1%), living with partners = 123 (61.8%), education = mean 14.6 (SD, 2.5) yrs SES (employed) = 132 (66.3%) & income > $50,000 = 83 (45%), parity= NR	N = NR, BW = 397- 4,706g, GA= 23-41 wks
**Trumello 2019, Italy**	Cohort prospective,1 centre Chieti University Hospital, NNU, study period & length of stay = NR	To explore psychological functioning and mental representations in mothers of preterm infants during NNU stay	Mothers to babies < 37 wks, mother's age ≥18 yrs, mother's good knowledge of the Italian language, and absence of mother's drug or alcohol addiction	Babies genetic illnesses, neonatal deformities, and neurological damages clinically identifiable at birth	N = 62 mothers, mean age 33.98 (SD, 4.76) yrs, all white, SES (middle) = 79%, parents lived together = 59 (95%), employed = 50 (80.6%), nulliparous = 43 (69.4%), university = 20 (31.8%)	N = NR, GA <32 wks = 40 (35.5%), ≥32 wks = 22 (64.5%), BW = mean 1685.42 (SD, 525.4)g

Abbreviations: NR: Not reported; BW: Birth weight; wks: Weeks; hrs: Hours, yrs: Years, GA: Gestational age; NICU: Neonatal intensive care unit, PRI: Perinatal risk inventory; SES: Socioeconomic status, HIV: Human immunodeficiency virus, SD: Standard deviation

a Studies included in both anxiety and PTS

The eligibility criteria differed across the included studies. Gestational age (GA) at birth was a specified inclusion criterion in 12 studies, published in 13 records^24^^,^^25^^,^^27^^,^^28^^,^^32^^,^^33^^,^^38^, ^39^, ^40^, ^41^^,^^43^, ^44^, ^45^ but GA criteria differed across studies: <30 weeks;^43^^,^^44^ <32 weeks;^27^^,^^32^ <33 weeks;^38^^,^^39^ <34 weeks;^41^ <37 weeks;^25^^,^^28^^,^^45^^,^^46^ 23 weeks to full-term .^24,36,37^GA was not reported in one study.^22^ Birthweight was also a specified inclusion criterion in five studies, but the birthweight criteria differed across studies: <1500g;^28^^,^^33^ <1700g;^34^ <1800g.^24^^,^^40^ Parents of babies who were less likely to survive and/or who had congenital anomalies, parents who were not fluent in a specific language, used recreational drugs, were HIV positive, had existing mental health conditions, or were aged <18 years were excluded across the majority of the studies.

### Risk of bias assessment

The risk of bias assessment for all studies reporting prevalence of anxiety can be found in Appendix C.^19^ No study had low risk of bias across all items.

### Anxiety Prevalence

Table 2 shows anxiety prevalence by assessment tool used in each included study. Clinical interviews were used in one study,^33^ self-report scales were used in 19 studies published in 21 records,^22^, ^23^, ^24^, ^25^, ^26^, ^27^, ^28^, ^29^, ^30^, ^31^, ^32^^,^^34^, ^35^, ^36^, ^37^^,^^40^, ^41^, ^42^, ^43^, ^44^, ^45^ and both clinical interview and self-report scale were used in one study reported in two records.^38^^,^^39^ Six different self-report scales were used to assess anxiety, most commonly the State-Trait Anxiety Inventory (STAI). Even when the same self-report scale was used, there was variation in the cut-off points reported.

**Table 2:** Anxiety prevalence data by time of assessment and assessment tool.

**Study ID**	**Assessment tool & cut-off**	**Assessment time**	**Gestational age (weeks)**	**Participants**	**N**	**n**	**%**
Time of assessment ≤1 month							
Clinical diagnosis							
Misund 2014	Clinical diagnosis	4–30 days after birth (median=11 days)	<33	Mothers	29	*5*	17.0
State-Trait Anxiety Inventory (STAI)							
Holditch-Davis 2015^a^	STAI State >47 (from author)	During admission	Mean 27.2 (SD 2.9)	Mothers	232	133	*57.3*
Dantas 2012	STAI State >40	During admission	26-37	Mothers	60	49	81.7
Greene 2015 & 2018^a^	STAI >40	2-4 weeks after birth	23.2-32.3	Mothers	69	38	55.0
Onay 2021	STAI State >40	≥7 days after admission	< 37 to 40	Mothers	93	48	51.6
Ong 2019	STAI State >40	≤48 hours of admission	27-34	Mothers	180	153	85.0
Trumello 2018	STAI State > 39	1 week after birth	< 32 to <37	*Mothers*	*62*	*39*	*62.0*
			<32	Mothers	40	*29*	72.0
			≥32	Mothers	22	*10*	45.0
Misund 2014 & 2016^a^	STAI State >39	2 weeks after birth	<33	Mothers	29	*20*	69.0
Harris 2018^a^	STAI State >33	Before discharge	≤32	Mothers	37	12	32.0
Cajiao-Nieto 2021	STAI >28 (from author)	3 days after birth	<37	Fathers	51	17	33.0
		6-18 days after birth		Fathers	51	5	10.0
Garfield 2015^a^	STAI State >20 (from author)	3 months after birth (60% =<1 month)	<37	Mothers	113	31	27.0
Hospital Anxiety and Depression Scale (HADS)							
Carter 2007	HADS ≥12	≤3 weeks after admission (mean=17 days SD=11.2 days)	23-42	Parents	*299*	*55*	*18.4*
				Mothers	119	35	18.0
				Fathers	180	20	11.0
Mulder 2014	HADS >11	During admission	23–42	*Parents*	*447*	*55*	*12.0*
				Mothers	242	35	18.0
				Fathers	205	20	11.0
Eutrope 2014^a^	HADS ≥8	1-5 days after birth	<32	Mothers	100	*75*	75.0
		15 days before discharge	<32	Mothers	*93*	*47*	*50.0*
Pace 2016^a^	HADS ≥8	2 weeks after birth	<30	*Parents*	*214*	*102*	*48.0*
				Mothers	113	55	48.0
				Fathers	101	47	47.0
Beck Anxiety Inventory (BAI)							
Segre 2014 & McCabe-Beane 2018	BAI ≥16	2 weeks after birth	23-41	Mothers	190	53	27.9
Hamilton Anxiety Rating Scale (HAM-A)							
Gonzalez-Hernandez 2019	HAM-A ≥18	2 weeks after birth	<37	Mothers	188	64	34.0
Zung Self-Rating Anxiety Scale (SAS)							
Kong 2013	SAS >50	6 days after admission	36.63 ± 3.34 (mothers) 37.09 ± 3.16 (fathers)	Parents	*600*	*128*	*21.0*
				Mothers	200	48	24.0
				Fathers	400	80	20.0
Depression Anxiety and Stress Scale (DASS)							
Das 2021	DASS≥21 (from author)	During admission	29-39	Mothers	96	37	39.0
Bonacquisti 2020	DASS>21	During admission	Not reported	Mothers	127	*23*	17.8
Assessment time >1 month ≤12 months							
Clinical diagnosis							
Structured Clinical Interview for DSM Disorders (SCID)							
Helle 2016^a^	SCID	4-6 weeks after birth	<37	Parents	*189*	*13*	*6.9*
				Mothers	111	*11*	9.9
				Fathers	78	*2*	2.9
STAI							
Greene 2015 & 2018	STAI >40	After discharge	23.2-32.3	Mothers	64	23	36.0
Rogers 2013	STAI >40	At time of discharge	<30	Mothers	73	31	43.0
HADS							
Pace 2016	HADS ≥8	6 months after birth	<30	Parents	*155*	*35*	*23.0*
				Mothers	81	*20*	25.0
				Fathers	74	*15*	20.0
Assessment time >12 months							
STAI							
Misund 2016	STAI >39	18 months corrected age	<33	Mothers	27	0	0.0

Abbreviations: N: Total sample, n: Number of cases; Italics: Numbers were calculated.

a Studies measured both anxiety and PTS.

### Anxiety Prevalence up to one month after birth

**Figure 2:**
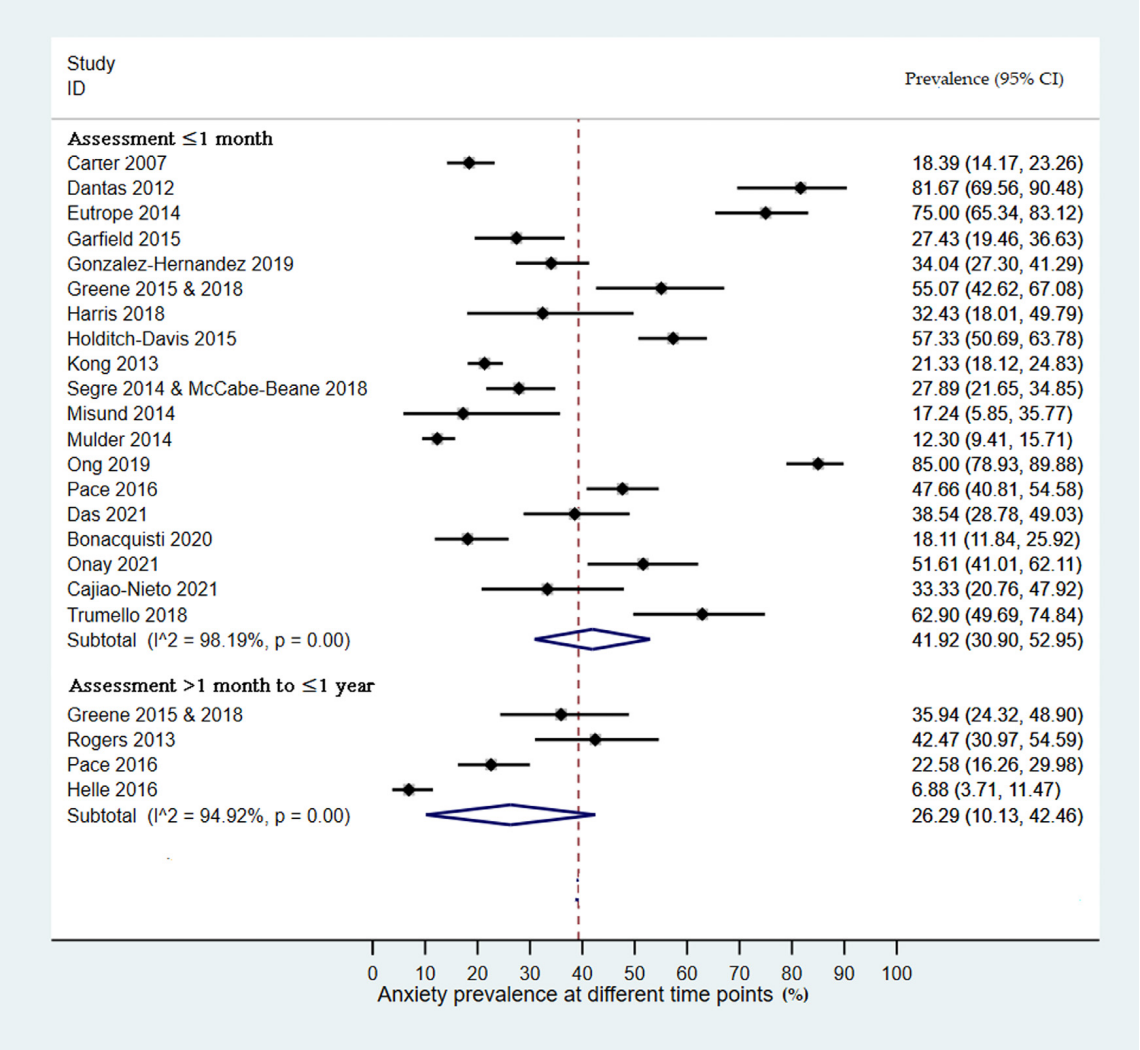
Forest plots of anxiety prevalence (%, 95% CI) among parents of babies admitted to NNU assessed ≤ 1 month and > 1 month to ≤ 1 year after birth. Subtotal is the pooled prevalence at each assessment time point; 95% CI is the 95% Confidence Intervals for the anxiety prevalence; I^2 represents the quantity of heterogeneity (0-100%); p is the p-value of the heterogeneity test.

The pooled prevalence and subgroup analyses for anxiety are shown in Table 3 and in forest plots in Figure 2 and Appendix D. The estimated pooled prevalence of anxiety up to one month after birth was 41.9 % (95%CI: 30.9, 53.0) across 19 studies including 3,377 participants. Individual study prevalence estimates ranged from 12.3-85% and there was high between-study heterogeneity (I^2^=98.2, p=0.00). Sub-group analyses showed higher prevalence of anxiety in mothers 42.3 % (95%CI: 30.7, 54.0) compared to fathers 22.9% (95%CI: 13.1, 32.8) and when STAI-state was used 52.3% (95%CI: 39.7, 67.9) compared to other scales 32.2% (95%CI: 21.1, 42.4). Univariate meta-regression analyses found evidence that sex of parent (β = 0.54: 95%CI 0.30 - 0.99, p = 0.041), and assessment tool (β = 0.65, 95%CI 0.41 – 0.96, p = 0.036) explained between-study heterogeneity.

**Table 3:** Pooled prevalence and subgroup analyses of anxiety.

**Outcome measure**	**Study (N)**	**Sample (N)**	**Prevalence(95%CI)**	**I^2^%**
Anxiety ≤one month	19	3,377	41.9 (30.9, 53.0)	98.2
Study setting				
High-income countries	14	2,256	36.0 (25.5, 46.5)	96.8
Middle-income countries	5	1,121	54.7 (24.8, 84.5)	99.0
Study design				
Cohort	13	2,226	40.8 (25.6, 56.0)	98.5
Cross-sectional	6	1,151	41.1 (25.3, 54.0)	96.7
Selection bias- Sample representativeness				
Representative	6	1,398	48.7 (27.3, 70.1)	98.3
Non-representative	13	1,462	37.4 (24.7, 50.0)	97.5
Anxiety symptoms				
Self-reported	15			
Parents*				
Mothers	19	2,708	42.3 (30.7, 54.0)	97.6
Fathers	6	669	22.9 (13.1, 32.8)	93.39
Prematurity ^a^				
GA 23 - 41 weeks	11	2,372	37.3 (27.5, 47.2.6)	96.6
GA <33 weeks	8	878	50.0 (34.9, 66.0)	96.0
Measuring scales*				
STAI-State b	9	892	52.3 (39.7, 67.9)	96.3
Other scales	9	2,456	32.2 (21.1, 42.4)	97.1
Anxiety > 1 month < 1 years	4	481	26.3 (10.1, 42.5)	94.9
Study Setting	4 in high-income countries			
Study design*				
Cohort	3	292	33 (20.1, 45.8)	NA
Cross-sectional	1	189	6.9 (3,71, 11.5)	NR
Selection bias - Sample representativeness*				
Representative	1	189	6.9 (3.71, 11.5)	NA
Non-representative	3	292	33 (20.1, 45.8)	NR
Anxiety symptoms*				
Clinical interview	1	189	6.9 (3.71, 11.5)	NA
Self-reported	3	292	33 (20.1, 45.8)	NR
Parents*				
Mothers	4	329	27.7 (12.0, 43.4)	91.6
Fathers	2	152	4.8 (1.6, 8.1)	NR
Prematurity*				
GA < 37 weeks	1	189	6.9 (3.71, 11.5)	NR
GA <33 weeks	3	292	33 (20.1, 45.8)	NR
Scales*				
STAI-State	2	137	39.3 (31.2 to 47.5)	NR
Other scales	2	344	10.5 (7.3, 13.7)	NR
Anxiety > 1 year	1	27	0	NA

Abbreviations: ^a^ Bonacquisti 2020 not included as gestational age (GA) not reported & Trumello 2018 provided data for both subgroups.

b Misund 2014 not included; *p<0.05: Significant difference between subgroups; NR: Not reported -few studies to calculate heterogeneity; NA: Not applicable.

### Anxiety prevalence from one month up to one year after birth

The estimated pooled prevalence of anxiety from one month up to one year after birth was 26.3% (95%CI: 10.1, 42.5) across four studies, published in five records, including 481 participants (Table 3 and in forest plots Appendix D). Individual study estimates ranged from 6.9% - 42.5% and there was high between-study heterogeneity (I^2^= 94.9 %, p = 0.00). Subgroup analyses showed higher prevalence in cohort studies 33% (95%CI: 20.1, 45.8) compared to cross-sectional studies 6.9% (95%CI: 3.71, 11.5), self-reported symptoms 33% (95%CI: 20.1, 45.8) compared to clinical interviews 6.9% (95%CI 3.71, 11.5), in mothers 27.7% (95%CI: 12.0, 43.4) compared to fathers 4.8% (95%CI: 1.6, 8.1), when GA < 33 weeks 33% (95%CI: 20.1, 45.8) compared to GA 32 -41 weeks 6.9% (95%CI: 3.71, 11.5), and when anxiety was assessed by the STAI-State 39.3% (95%CI: 31.2 to 47.5) compared to the others scales 10.5% (95%CI: 7.3, 13.7) (Table 3). Meta-regression was not performed due to insufficient numbers of studies.

### Anxiety prevalence more than one year after birth

Only one study, reported in two records,^38^^,^^39^ including 27 participants assessed prevalence of anxiety more than one year after birth. No participant in the sample scored above the cut-off for clinically significant symptoms.

### Post-traumatic stress

A detailed description of the included studies is provided in Table 4. In total 35 studies, published in 41 records, involving 3,380 parents. Eleven studies, published in 14 records, involved mothers and fathers;^46^, ^47^, ^48^, ^49^, ^50^, ^51^, ^52^, ^53^, ^54^, ^55^, ^56^, ^57^, ^58^, ^59^ one study involved fathers only [60] and the remaining 23 studies, reported in 26 records, involved mothers.^27^^,^^28^^,^^30^, ^31^, ^32^^,^^34^^,^^38^^,^^39^^,^^60^, ^61^, ^62^, ^63^, ^64^, ^65^, ^66^, ^67^, ^68^, ^69^, ^70^, ^71^, ^72^, ^73^, ^74^, ^75^, ^76^, ^77^, ^78^

**Table 4:** Characteristics of included studies - PTS.

**Study ID, country**	**Study design &setting, Study period, Neonatal unit type of care, length of stay**	**Study objective**	**Study inclusion criteria**	**Study exclusion criteria**	**Parents’ characteristics**	**Babies’ characteristics**
**1. Aftyaka**						
Aftyka 2014, Poland	Cross-sectional, 1 centre in Lublin, July 2012 to October 2014, NNU - level III mean length of stay mothers = 21.2 (SD, 25.3) days, fathers = 24.8 (SD, 29.2) days	To determine the frequency and medical, demographic risk factors for PTSD in parents of NNU neonates	Mother and fathers of infants who were hospitalised in NNU	Inability to read and write in Polish, lack of consent to participate in the project and taking care of a child by legal guardians who were not the parents	N = 39 mothers, N = 27 fathers. mothers age = mean 31.0 (SD 5.4) yrs, fathers age = 32.8 (SD 4.6) yrs, higher education: mothers = 16 (46%) vs. fathers = 14 (64%), nulliparous 21 (54%) = first born fathers 13 (50%), SES = mothers employed 16 (46%) vs fathers 14 (64%); parity ethnicity & relationship = NR	N = 42, GA = infants of mothers = 34.3 (SD, 4.8) wks; BW = 2362 (SD, 1050)g, included babies with congenital malformations
Aftyka 2017 & 2020, Poland	Cross-sectional, 1 centre in Lublin, the only paediatric university hospital in south-eastern Poland July 2012 to October 2014, NNU – level III, mean length of stay = mothers 21.6 (33.6) vs fathers 20.7 (23.4) days	To describe coping strategies and identify the potential risk factors related to basic sociodemographic and medical data as well as coping with the development of PTSD in the parents of NNU neonates	Biological parents of NNU babies, speak and write in Polish	Inability to read and write in Polish, lack of consent to participate in the project and taking care of a child by legal guardians who are not the parents	N =72 mothers, N = 53 fathers, mothers age = 30.3 (SD, 5.1), fathers age = 32.3 (5.2) years, living with partner = 49 couples, party, ethnicity, education, SES = NR	Mothers N = NR, GA = 34.74 (SD, 4.67) wks, BW = 2,407 (SD, 963)g, fathers: N = NR, GA = 34.60 (SD, 5.05) wks, BW = 2,377 (SD, 1025)g
**2. Ahlund** **2009, England**	Cross-sectional study, 1 centre Norfolk & Norwich University Hospitals, Babies born in 2004, questionnaire sent in 2006; NNU, length of stay = NR	To determine the prevalence of symptoms of PTSD 2–3 years after delivery in mothers who had given birth to VLBW infants <1500g	Mothers of VLBW<1500g alive babies, identified from neonatal register	Not alive infants at the assessment time	N = 70 mothers eligible, N= 24 responded, other characteristics: NR	N= NR, BW = median 1,120 g (range= 634–1,490)g; GA < 37 wks, median 30.2 wks (range 24–34)
**3. Barr 2010, Australia**	Prospective cohort study, 1 centre, Royal Alexandra Hospital for Children, December 2007 through November 2008, NNU, referral center for newborn infants with complex medical, cardiac, and surgical diseases, length of stay => 4 days	To explore the relation between personality predisposition to shame, and fear of death of parents of NNU infants	English literate, infant was≥ 34 wks at birth, treated in the NNU for at least 4 days, alive when the study questionnaires were completed	Not alive infants at the assessment time	N= 204 parents, 110 mothers and 94 fathers, mother's age, mean (30.2) SD 5.64 vs. father's 33.4(6.63) yrs, Parents Oceanian (68%), European (12%), Asian (9%), Middle Eastern (8%), and other (3%), (37%), university degree (30%), living with partner: 96%; parity & SES: NR	N= NR, GA ≥34 wks, BW= NR
**4. Chang 2016, Taiwan**	Cross sectional, 1 centre, January 2010 to June 2011, NNU, length of stay <60.00 ± 53.78 days	To estimate the prevalence of symptoms of distress in mothers of preterm NNU infants and factors complications of delivery for these symptoms	Parents to babies <37 weeks gestation, admission to the NNU, and infant survival at the time of the interview	Not understanding Chinese, refused to consent, babies with congenital chromosomal abnormalities/congenital defects, significant heart disease after birth, or died during the hospital stay or after leaving the NNU, mothers with major illnesses, cancer, or psychiatric disorders	N = 102 mothers, mothers mean age = 34.28 (SD, 4.45), Nulliparous: (36.27%), education: > 12 years = 95 (94.14%), SES household income of ≦600,000 NTD (about 19,679 USD) = 52 (50.98%); ethnicity & relationship status = NR	N = NR, GA = 31.53 (SD, 2.97) wks, BW = 1661.86 (SD, 563.82)g
**5. Clark 2021, USA**	Cross-sectional study, 1 Midwestern centre, July 2009 - July 2014, NNU level IV, length of stay = NR	To characterise the experience of bereaved parents of babies admitted to NNU	Parents of infants who died within the previous five years in level IV NNU	Age <18 years, infants died within the past 3 months, not speaking English	N = 40 mothers, mean age = 33.33 yrs, white = 35 (88%); living with partner = 32 (80%); education (secondary) = 34 (85%); N = 27 fathers mean age 36.74 yrs, white = 16 (58%); living with partner = 24 (60%), education (secondary)= 18 (67%), SES & parity = NR	N, BW & GA = NR
**6. Eutrope 2014**^a^**, France**	Prospective cohort, 3 centres in Reims, Besancon and Nancy, January 2008 and January 2010, NNU, length of stay = NR	To describe precocious maternal feelings when the mother has to face the premature birth of her infant	Mothers to infants < 32 wks	For mothers psychiatric illness, drug or alcohol abuse, aged <18 yrs, language barriers; for newborns = unfavourable vital prognosis evaluated Perinatal Risk Inventory score >=10 infants risk of significant developmental disabilities and malformation and/or genetic anomaly diagnosed	N= 100 mothers at visit one, N= 93 at visit two, mean age 29.8 (SD,6.0), range 17– 45 yrs, 92% lived with partners, education = higher education 79.29%, SES = 69% work, nulliparous 48%,ethnicity = NR	N=100 visit one, visit two N= 93, GA < 32 wks, mean BW = 1320g, all singleton
**7. Feeley 2011, Canada**	Prospective cohort, 2 centres University Teaching Hospital in Montreal, study period = NR, levels 3B (provides mechanical ventilation no major surgery) or 3C (provides major neonatal surgery but neither open-heart surgery nor extracorporeal membrane oxygenation, length of stay = NR	To examine mother's PTSD symptoms in relation to mothers and infants’ characteristics and to the quality of mothers' interaction with infants & their development	Mothers to NNU infant with BW < 1,500 g, GA < 32 wks speak English or French, and lived within 1-hour drive from hospital	Babies with congenital anomalies, sensory/motor disability	N = 21 mothers, age = 21 to 41, mean 30.9 SD,5.4) yrs, all lived with partners & house wives, nulliparous = 71.4% (n = 15). education: 11 -18 (M = 15. 2, SD = 1.9) yrs, ethnicity North American born (n = 12, 57.1%), 9 (42.8%) born elsewhere, 3 women (14.3%) had resided in Canada <1 yr	N= 21,GA < 32 wks, singletons, 29% (n = 6) twins or triplets, BW&GA= NR
**8. Forcada-Guex 2011, Switzerland**	Cohort, 1 centre at University of Lausanne Hospital, January to December 1998, NNU Level 3, length of stay = NR	To clarify links between maternal traumatic reactions, quality of mother–infant interactions and maternal representations of infant's attachment	Mothers to preterm baby GA < 34 wks	Infant malformation, chromosomal abnormalities and fetopathy, parents’ psychiatric illness and/or drug abuse, not speaking French	N= 47 mothers nulliparous = 30 (64%), living with partner = 39 (83%) nationality (Switzerland & EU) = 44 (94%), SES* = 2.4 (0.6%), education= NR	N = 47, GA mean = 31 wks (SD, 2), singleton = 37 (79%), BW = NR
**9. Garfield 2015**^a^**^,^ USA**	Cross-sectional; 2 hospitals inner-city medical centres serving underserved and uninsured populations, study period, NNU level & length of stay = NR	To identify risk factors among urban, low-income mothers, to enable NNU healthcare providers more effectively screening and referral	Mothers of VLBW<1500 g and preterm < 37 wks, English speaking, no current mental health diagnosis, infants clinically stable and did not have a congenital neurological problems or symptoms of substance abuse	< 18 yrs old, ongoing critical illness (HIV, seizure), major depression, psychosis, bipolar disease; mothers to infants receiving mechanical ventilation	N = 113 mothers, mean age = 24.7 (SD = 5.17) yrs, ethnicity African American = 81%, living with partner: 52.3%, education high school graduates = 43%, SES = 39% received public aid and an additional 40% were uninsured; parity =NR	N = NR, GA <37 wks, BW =mean 1073 (SD, 342)g
**10. Gateau 2021, USA**	Cross-sectional; 1 centre in Los Angeles, 2013 – 2015, NNU level & length of stay: NR	To report the prevalence acute posttraumatic stress & depression in low-income families after NNU discharge	One parent English- or Spanish-speaking to preterm infants who were up to 24 months corrected age with completed developmental assessments	NR	N = 139 mothers, age = NR, ethnicity = white 10 (1%), Hispanic 102 (73%), black & others: 25 (18%), education (college): 76 (55%); SES (< $20,000 income)= 82 (59%); parity& living with partner =NR	N =116; GA = < 24 - < 28 (n= 52), 28 - 33 (n=57), >34 - <37: (n=7); BW <1,500 (n = 85); BW ≥1500 -<2,500 = (n=16); BW ≥2,500 = (n=7)
**11. Goutaudier 2011, France**	Mixed method design, Quant-cohort; 1 centre; November 2009 to May 2010; NNU level & length of stay = NR	To assess mothers’ experience of preterm delivery and hospitalisation and psychopathological consequences	Mothers age >18 yrs, French speaking ad delivered preterm NNU babies	NR	N= 27, age 19–36 yrs, mean 29 (SD, 2.7), yrs CS: 15 (56%), other characteristics: NR	N= 27, GA = 27 -37 wks, mean= 30.6 (SD,2.7) wks, 22.2% born at 32–37 wks; 48.1% born at 28– 31 weka; 29.7% <28 wks, BW = NR
**12. Greene 2015 & 2018**^a^**, USA**	Prospective cohort; 1 urban centre; 2011-2012; NNU level IV, length of stay: NR	To identify the associations between elevated maternal depression, anxiety and PPTS at two time points during the NNU hospitalization	English-speaking mothers, >18 years, babies more likely to survive and VLBW<1500g	Congenital anomalies, drug users	N = 69 at T1, N= 64 at T2, age = 27 (SD, 6) yrs, nulliparous 23 (34%), ethnicity black = 38 (54%), Non-Hispanic white = 18 (26%), Hispanic = 12 (17%), living with partner = 32 (51%); education: highest grade completed mean = 13.4 (SD, 2.4)	N = 69, GA = 27.5 (SD, 2.2) wks, range 23.2- 32.30 wks; BW = 957(SD, 243)g
**13. Harris 2018**^a^**, USA**	Cohort study; 2 centres Saint Louis Children's Hospital & Barnes Jewish Hospital's Special Care Nursery, January to June 2015, NNU Level III & IV length of stay = 83.4 (40.9) days	To examine early mental health challenges in mothers of VPT in NNU and mothers of full-term babies, to factors associated with high levels of maternal psychological distress	Mothers who had an infant born = <32 weeks GA & no congenital anomalies	Drug use mothers	N=37 mothers, age = 29.7 (6.4) yrs, multiple birth 5 (13%), SES (<$25,000) = 16 (43%), college degree 15 (41%), single 7 (19%)	N= 50, GA ≤32 wks, BW = mean 1104.0 (SD, 416.7)g
**14. Helle 2018**^a^**, Germany**	Cross-sectional study, 3 largest perinatal medical centres in Hamburg, 2006 – 2008; NNU, level & length of stay = NR	To investigate the level of postpartum PTS, prevalence of and risk for postpartum PTSD and Acute Stress Disorder in both parents with a preterm VLBW infant compared to parents with term infants and identifying predictors for postpartum PTSS	VBW <1500g, PT<37 wks GA	Insufficient German skills, inability to follow study procedures, premature discharge, residing too far from the study centre, infant died before the first assessment	N = 111 mothers, N = 78 fathers, mothers mean age = 32.6 (SD, 4.7) yrs, nulliparous = 82 (73.9%), living with partner = 109 (98%), SES Low = 11 (10%), ethnicity & education	N = NR, GA = 28.2 (SD, 2.7), BW = 1095.9 (SD, 330.4)g, singleton birth = 76 (68.5%), twin - 32 (28.8%), triplet = 3 (2.7%)
**15. Holditch-Davis 2009, USA**	Cohort longitudinal repeated measures design as part of RCT - NNUs of 2 hospitals in one state, study period, NNU level & length of stay = NR	To examine inter-relationships among stress due to infant appearance and behaviour in the NNU exhibited by African American mothers of preterm infants	African American biological mothers of preterm infants < 1500 gm at birth or requiring mechanical ventilation. Mothers were recruited when their infants were no longer critically ill	Infants with congenital, symptomatic from substance exposure, hospitalized > 2 months post-term, or triplets or part of a higher order multiples set; mothers with no custody, follow-up for 2 years unlikely, HIV+, < 15 yrs, critically ill, not speak English, mental health problems	N=177 mothers, mean age=25.9 (SD, 6.5) yrs, living with partner = 6.1%., mean education =12.6 years (SD, 1.8); SES Public assistance =52.8%; ethnicity: all African American, parity = NR	N= NR; mean GA=28.3 (SD,2.9)wks; mean BW=1107 (SD,394)g
**16. Holditch-Davis 2015**^a^**, USA**	Cohort, longitudinal repeated measure design as part of a randomized controlled trial - NNUs of 4 hospitals in two states, study period, NNU level & length of stay = NR	To estimate the inter-correlations between depressive symptoms, state anxiety, PTS, stress due to infant appearance and behaviour, and stress due to parental role alteration in a multi-ethnic sample of m others of preterm infants during initial hospitalization	Mothers of PT babies of BW< 1750g	Parents to infant with congenital neurological, symptoms of substance exposure, age< 15yrs; HIV+; psychosis/bipolar disease; depression, critical illness; non-English speaking), or follow-up for 12 months was unlikely	N= 232 mothers, age mean =27.0 yrs (SD,6.1), living with partner= 32.3%, mean education =13.4 yrs (SD,2.3), ethnicity white=19.8%, black=69.8%, Hispanic=8,1%, other=1.9%. nulliparous=55.1%., SES= Public assistance=20.3%	N= NR; mean GA=27.2 wks (SD,2.9); BW= mean1006.2 (SD,326)g
**17. Jubinville 2012, Canada**	Prospective cohort, 1 centre in Alberta; February - May, 2008; NNU, level III, length of stay = NR	To determine whether significant symptoms of (Acute Stress Disorder) are present in mothers of premature NNU infants	Mothers of infants’ < 33 weeks GA admitted to NNU	Infant with foetal anomaly, severe illness requires compassionate care and/ or maternal illness precluded NICU visit and assessing women at 7 -10 days after birth	N= 40 mothers, mean age 29.2 (SD, 5.8) yr, education above high school = 24 (60%), high SES (income =$60 000 per year = 23(58%), living with partner = 37(93%), majority white n = NR	N= 52, 10 twins, & one triplets, BW mean 1374.5 (SD, 466.1)g, rang 640-2220 g; GA =mean 29.0 (SD,2.6) wks, range=24.0-32.0) wks
**18. Koliouli 2016, France**	Cross-sectional, 1 centre at University Hospital of Toulouse, January 2013 - March 2014; NNU level = NR, length of stay = Postmenstrual age at discharge = 39.8 wks (SD, 5.2)	To explore the feelings of stress, PTSD, and the coping strategies of fathers of premature infants	French-speaking fathers of preterm infants GA < 35 wks	Fathers to infants with congenital problems	N= 48 fathers, mean age 33.5 (SD = 3.5) yrs, all living with partner, 91.5% French, 51.1% University degree, SES = 37.2% intellectual profession	N= 48, 52.5% born at GA 26-28 wks, 47.5% at 29-35 wks
**19. Lefkowitz 2010, USA**	Prospective cohort, 1 large eastern United States Children Hospital, 9 months period, NNU level IV, length of stay = 91 days (SD, 37.1) days	To assess the prevalence and correlates of (Acute Stress Disorder) and (PTSD) in mothers and fathers, and postpartum depression (PPD) in mothers, of NNU	Mothers and Fathers of infants on NICU who were anticipated to stay on NNU >5 days	Inability to read English, parent age <18, or if the child's death appeared imminent	N= 89 mothers, N=41 fathers, mean mothers age = 29 yrs vs 33 yrs for fathers, ethnicity = Caucasian mothers 71% vs. 81% fathers, education college degree 24.4%, mothers vs 21.4%. fathers, relationship status & SES = NR	N = NR, GA < 30 wks
**20. Lotterman 2019, Columbia**	Cohort study, 1 centre Morgan Stanley Children's Hospital, Columbia University Medical Centre, NNU level III& IV; length of stay 83.4 (SD,40.9) days, study period = NR	To explore whether mothers of moderate- to late-preterm infants had elevated rates of psychological symptoms	Mothers of moderate- to late-preterm infants	Mothers to babies born <32 wks or later than 36 weeks, or if they had been in the NNU for longer than 6 months	N=91 mothers at NNU admission, N = 76 (83.5%) at 6 months, mean age = 32.45 (SD, 6.78) yrs, ethnicity = 40.7% Caucasian, 38.9% Hispanic 17.4% African American, 10.5% Asian, 2.3% American Indian/Alaskan Native, 29.1% other, mean years of education =14.29 (SD,4.30) yrs, living with partner = 86.6%; parity &SES= NR	N= NR; GA 32–37 wks, GA= mean 33.53 (SD, 1.33) wks, BW= NR
**21. Malin, 2020, USA**	Cohort study, 1 centre,; NNU – level IV, length of stay ≥14 days, study period = NR	To determine if PTSD among parents of NNU babies can be predicted by objective measures or perceptions of infant illness severity	Parent of infants who were in NNU ≥14 days	Parents did not speak English, infants discharged home with their non-biological parent, infant was previously discharged home/ transferred, infants who died in NNU	N= 164 parents; living with partner = 154 (94%), SES (government insurance) = 82 (50%); parity, ethnicity & education = NR	N = 164; GA = 23-28 wks (n=36), 29-33 wks (n=60), 34-36 wks = (n=29), >37 wks = (n=39); BW <1000g = (n=28); BW > 1000g = (n=136)
**22. Misund 2013, 2014 & 2016**^a^**, Norway**	Cohort study, 1 centre Oslo University hospital, June 2005 to July 2008 in two periods of measurement; NNU level & length of stay = NR	To explore long-term mental health outcomes in mothers experiencing preterm birth and to identify interactional, main effect variables and predictors	Mothers to preterm babies <33 wks admitted to NNU	Mothers of severely ill babies that the medical staff estimated to have poor chance of survival, and non-Norwegian speakers	N=29 mothers at 2 wks post birth, N=27 at 2 wks after NNU admission, N= 26 at 6 & 18 months post term, age: 33.7 yrs (4.3), 89.7% > 12 years education = 29 N= 26 at 18 months mean age =33.7 (SD, 4.3) yrs, nulliparous = 18 (62.1%) education≥12 years = 26 (89.7%); all living with partner, SES= 13.8% unemployed, nulliparous 62.1%, ethnicity= NR	N= 35, GA=29 median(range=24-32) wks median BW=1.2 kg (range=0.6-2.0); 40% twins
**23. Naeem 2019, Iran**	Descriptive-comparative study cohort, 2 hospitals (Yas and Vali-e-asr Hospitals); NNU, 2016 and 2017; length of stay = NR	To compare the prevalence of PTSD in parents of hospitalized preterm and term neonates	Parents of NNU preterm (GA 24 - 36 wks), and parents to hospitalized terms (GA >38 wks) Both aged 2 to 5 days	Parental psychiatric or underlying diseases, smoking and drug abuse	N=80 parents mothers vs. fathers: N=79 vs. 79, education: upper diploma 57 (72.2%) vs. 51 (64.6%), unemployed 67 (84.8%) vs. 6 (7.6%) full term: N=80 parents, mothers vs. fathers: N:80 vs 80, education upper diploma: 50 (62.5%) vs. 47 (58.82%) unemployed 69 (86.3%) vs. 4(5%)	PT: N=80; GA: GA 24 - 36 wks), FT: N=80, GA >38 wks
**24. Pace 2020**^a^**^,^ Australia**	Prospective cohort; 1 centre Royal Women's Hospital, Melbourne, January 2011 to December 2013; NNU level & Length of stay = NR	To report the proportion of parents of VPT infants with PTSS symptoms at different time points	Families with very preterm infants, GA <30 wks admitted to NNU	Parents who did not speak English, infants with congenital abnormalities, unlikely to survive	Mothers 89, Fathers 75v 92 mothers &/or 75 SES parents (high risk): 45 (43%) Mean mothers age: 33 (5.3) yrs; mothers education (>12 yrs): 62 (67%); fathers age: 35 (6.2) yrs; fathers education (> 12 yrs): 45 (60%)	N= 131; GA < 30 wks; mean GA 27.8 (1.5) wks; mean BW 1038 (261)g
**25. Pierrehumb** **2003, Switzerland**	Prospective cohort,1 centre Lausanne University Hospital; January to December 1998, NNU; level & length of stay= NR	To examine the effects of post-traumatic reactions of the parents on sleeping and eating problems of the children	Preterm infants <33 wks; infants were grouped into low and high risk based on perinatal risk inventory, basis of perinatal factors such as the Apgar score, gestational age, weight, head growth, electroencephalogram, ultrasonogram, and ventilation	Infant malformation, chromosome abnormality, and fetopathy; parental psychiatric illness and/or drug abuse, not speaking French	Low risk N= 23 mothers, N = 18 fathers, mothers age = 30.9 (SD, 4.3), fathers age = NR, parity = 0.45 (SD,0.59), single mother: 0/23. High risk N = 28 mothers, N = 23 fathers, mothers age = 31.3 (5.0), fathers age = NR, parity = 0.81 (SD, 1.24), relationship status: single mother = 1/27, ethnicity, education & SES = NR	Low risk babies: N =23 (GA = 31.3 wks (SD,1.5), BW = 1615g (SD, 280), High risk: N =27; GA = 24/27 (89%); BW = 1131 (SD, 318)g
**26. Rodriguez 2020, Argentina**	Cohort study; 1 centre, March 3, 2014-November 22, 2016; NNU level & length of stay = NR	To detect PTSD frequency and symptoms among mothers of VLBW preterm < 32 wks	Mothers with singleton pregnancies to VLBW (<1,500g) preterm babies (<32 weeks)	Mothers with psychiatric disorders before and/or during gestation, babies with chronic conditions & congenital malformations	N = 146 mothers, age ≤21 to ≥ 42 years, other characteristics = NR	N =146, GA < 32 wks, BW < 1,500g
**27. Sharp 2021, USA**	Cross-sectional – survey study via social media, November 2015 - July 2016, number of centres & NNU level = NR	To report on maternal perceived stress to infants’ NNU admission and the relationship between traumatic childbirth and PTSD	Biological mothers =>18-years-old, USA residents, complete the survey in English, alive infants age 1-4 months	Completing < 75% of the survey, infants age > 1-4 months	N = 77 mothers, mean age =39.6 (5.8) yrs; Ethnicity = White: 68 (88.3%); Hispanic: 7 (9.1%); living with partner: 73 (94.8%); Education (Bachelor's degree or above) = 35 (45%); SES (unemployed) = 26 (47%); Nulliparous = 32 (41.6%)	N= NR, BW <2,500g = 47 (61.0%), GA < 37 wks= 43 (55.8%)
**28. Shaw**						
Shaw 2006, USA	Prospective cohort, 1 centre, NNU, study period, NNU level & length of stay = NR	To examine the prevalence of (Acute Stress Disorder) in parents of NNU infants	English-speaking parents to infants in NNU	NR	N = 40, 24 couples,13 mothers, 3 fathers; mothers mean age = 33.96 years, ethnicity Caucasian (60%), living with partner = 87%, education B.A/B.S (72%), fathers mean age 37, ethnicity Caucasian (92.3%), living with partner(100%), education higher (41.7%), SES = family income > $80,000 a year 87.2%), parity=NR	N = NR, GA mean 31.46 wks (SD, 4.91) wks, BW mean 1,811.44 (SD, 986.97)g
Shaw 2009, USA (Follow-up of 2006)	Prospective cohort, 1 centre, NNU, mean hospital stay 12 (SD, 8) days, study period = NR	To describe the early-onset symptoms of Acute Stress DISORDE in parents and factors related to PTSD, identifying high-risk parents who may benefit from early intervention	English-speaking parents of NNU infants	NR	N = 18, N = 11 mothers, N = 7 fathers, mothers age = 34.55 (SD, 4.41) yrs, fathers age = 36.57 (SD, 4.79) yrs. parity = NR, ethnicity: mothers white = 7 (63.6%), Asian = 3 (27.3%), fathers white = 6 (85.7%), Asian = 1 (14.3%), fathers & mothers all living with partner, education mothers higher 10 (91%) vs 6 (86%) fathers: full time job fathers 100% vs 60% mothers, parity = NR	N = NR, GA = 30.89 (SD, 4.11) wks, range (27 to 41) wk, BW mean = 1,664.39 (SD, 908.21)g, range (1052-4004)g
**29. Shaw 2014, USA**	Cross-sectional, Lucile Packard Children's and El Camino Hospitals in northern California, July 2011 and December, 2012, highly specialized NNU level & length of stay = NR	To determine whether there are easily identifiable maternal socio-demograp; hic characteristics, aspects of their pregnancy history or factors related to their infant's medical history in postpartum mothers who screen positive for symptoms of psychological distress	English- and Spanish-speaking mothers of infants born between 26 and 34 weeks, weighing >1000 g, likely to survive	Psychiatric risk factors including suicidal or homicidal ideation or the presence of psychotic symptoms, for babies no major health complications such as congenital abnormalities	N = 135, age = 31.4 (SD, 5.5), nulliparous = 16 (53.3%), ethnicity white 19 (63.3%), black 0 (0%), Asian 10 (33.3%), other = 1 (3.3%), Hispanic = 13 (43.3%), living with partner = 29 (96.7%), education postgraduate degree = 11 (36.7%), SES: <$50k = 9 (30%)	N=NR; GA= 26-34 wks, BW = NR
**30. Schecter 2020, USA**	Cross-sectional, 2 centres NNUs at 1 hospital in Long Island, NNU level II & IV, study period & length of stay = NR	To investigate whether (PTSD) symptoms exist >1 year after neonatal intensive care unit (NNU) experience and if PTSD symptoms differ across parents of infants of different gestational age	Parents of infants attending a follow-up appointment were eligible regardless of the infant's GA or medical diagnoses	NR	N = 91 mothers and fathers, only 83 individuals identified their race: 41% white, 16% Hispanic/Latino, 15% black, 13% Asian, 6% multiracial, and 9% other, SES = 33% lower than the median, age, parity, education & living with partner = NR	GA < 28 wks 21%, 28 – 31 wks, 33%; 32 – 36 wks 38% and > 36 wks 9%, N & BW= NR
**31. Toly 2019, USA**	Descriptive correlational design, cohort study, 1 hospital, NNU transitional care unit in a large children's hospital located in the Midwest United States that has approximately 1000 admissions per year, study recruitment took place over a period of 15 months, NNU level= NR, length of stay Low risk = 40.7 (SD, 14.8) days High risk = 67.3 (23.7) days	To examine mothers’ psychological state prior to discharge of their technology-dependent infant from the NNU to home	Mothers > 18 years, their infant was to be discharged from the NNU to home within 2 to 3 weeks for the first time and was not dependent on medical technology (mechanical ventilation, intravenous medication, supplemental oxygen, tracheostomy, feeding tubes and they were able to read and speak English	Mothers of infants with a terminal diagnosis	N= 19, age range 18-41 years, mean 25.63 (SD, 6.27) yrs, ethnicity = 47.4% African American, 60% single, 58% high school, parity & SES = NR	N= NR, GA = 23- 39.29, mean 29.78 (6.43) wks, BW range 500-3765g, mean BW 1546.1g
**32. Vanderbilt 2009, USA**	Cohort, Boston Medical Center; study period, NNU level & length of stay = NR	To evaluate rates of acute posttraumatic stress symptoms and positive acute stress disorder screens among low-income mothers of infants admitted to the NNU compared with those with infants in the well-baby nursery	Mothers were identified from the daily census in the WBN or NNU at Boston Medical Centre and recruited based on availability of the research assistant, a living child, understands English, and having retained custody of the infant	HIV exposure or had pre-existing major mental illness. Infants and their mothers were excluded if the infants had substance withdrawal, major congenital anomalies, chromosomal abnormalities, foetal alcohol syndrome, cerebral palsy, blindness, or deafness to focus on a homogenous sample of NNU admissions with our limited sample size	N = 59 mothers mean age = 29 (SD, 6.8) yrs, ethnicity black = 35 (59%), Hispanic = 13 (22%), white 6 (10%), other 5 (9%), living with partner= 16 (27%), education high school/ below = 36 (61%), SES public insurance = 50 (85%), parity = NR	N = NR, GA mean 34 wks (SD, 3.8); BW= 2357(SD, 1034)g, twin = 3 (5%)
**33. Vinall 2018, Canada**	Cohorts, 1 tertiary-level NNU in Halifax, Nova Scotia, July 2012 and March 2016, NNU level=NR, length of stay mean 57.89 (SD,35.87) days;	To examine whether the number of invasive procedures together with mother's memory for these procedures were associated with PTSS at discharge from the NNU	mothers of infants < 37 weeks GA	Infants were excluded if they had major congenital anomalies, were receiving opioids, or underwent surgery	N = 36 mothers, age median age (IQR)= 31 (27-36) yrs, education median (IQR) = 5 (4-5) yrs, ethnicity, parity, education, relationship and SES = NR	GA median (IQR) 32 (30-34) wks, N, GA & BW= NR
**34. Yaman 2015, Turkey**	Cross- sectional, 2 centres, 2 January - 31 June 2012, NNU level=NR, length of stay 14 and 96 days, mean = 55.67 (SD, 28.54),	To examine the posttraumatic stress of mothers and fathers, the differences between their experiences	Parents of newborn in the NICU for at least 7 days, age > 18 yrs old, no previous experience of the NNU, no history of chronic diseases or psychiatric disorders	Parents who could not participate in the study	N= 66 couples, 40.9% of mothers 21–25-year age, 46.9% of fathers 26–30-year; 39.4% of mothers and 34.8% of fathers high school graduates, SES unemployed= 80.3% mothers vs. 92.4% fathers were working, nulliparous 60.4%	N= NR, 62.2% GA of 24–37 wks, 50% were age 8–28 days, 37.9% treated in the NICU for 8–28 days, 21.2% congenital anomalies
**35. Zerach 2015, Israel**	Cohort study, 1 hospital at Tel Hashomer, NNU level = NR, length of stay: NR (but according to hospital policy, the minimum length of stay for 24-27 weeks prematurity was 9 weeks)	To examine the relationship between extremely low birth weight (ELBW) children and their mother's stress and PTSD symptoms	Mothers of ELBW <1kg infants born at Tel Hashomer hospital from 1995 to 2006 and admitted at the centre's NNU	Mothers of infants who had died (N=2)	N= 78 mothers, mean age at data collection 39.53 (SD, 6.73) yrs; age at time of birth 29.89 (SD 5.76) yrs; 82.3% living partner, 53.2% educated to degree or higher, 34.6% above average income, parity = NR	N = 78 (75 ELBW, 3 VLBW) GA mean 25.5 (SD,0.71 wks), range 24 -27 wks multiple birth = 42.3% ELBW (<1kg): = 96.2% mean 752.67 (SD, 66.59)g, VLBW (<1.5kg) = 3.8% mean 1095.66 (SD, 110.21)

Abbreviations: PTSD: Post-traumatic stress disorder; NNU: Neonatal unit; GA: Gestational age; BW: Birth weight; SES*: Socio-economic status using Pirrehumbert 4-point scale; wks: weeks; NR: Not reported; HIV: Human immunodeficiency virus, EU: European Union; IQR: interquartile range; ELBW: Extremely low birth weight; SD: Standard deviation; VLBW: Very low birth weight; PT: Preterm, FT: Full term; yrs: Years.

a Studies included in both anxiety and PTS.

### Study characteristics

The eligibility criteria differed across the studies. Gestational age at birth was a specified inclusion criterion in 20 studies, published in 26 records,^27^^,^^28^^,^^30^, ^31^, ^32^, ^34^^,^^38^^,^^39^^,^^46^^,^^50^, ^51^, ^52^^,^^54^^,^^57^^,^^60^, ^61^, ^62^^,^^64^^,^^67^^,^^68^^,^^70^^,^^71^^,^^74^^,^^75^^,^^77^^,^^78^ but GA criteria differed across studies: < 32 weeks;^27^^,^^32^^,^^57^^,^^71^^,^^79^; < 33 weeks;^38^^,^^39^^,^^62^^,^^67^^,^^75^ < 34 weeks;^50^^,^^74^^,^^77^ < 35 weeks; ^60^ and < 37 weeks.^28^^,^^46^^,^^54^^,^^61^^,^^68^^,^^78^ Birthweight was an inclusion criterion across eleven studies, published in twelve records, but specific birthweight criteria differed across studies: < 1500g;^28^^,^^30^^,^^31^^,^^46^^,^^64^^,^^70^, ^71^, ^72^ < 1750g;^34^ <1000g.^76^ One study,^58^ included only parents of deceased babies. Common exclusion criteria were parents with existing mental health problems and parents of babies who were less likely to survive and/or who had congeniality anomalies.

### Risk of bias assessment

The risk of bias assessment for all studies assessing PTS ^19^ can be found in Appendix E. No study had low risk of bias across all items.

### PTS Prevalence

Table 5 shows the prevalence of PTS by assessment tool used in included studies and time of assessment. Clinical interview was used in two studies,^46^^,^^54^ clinical review of self-report scales was used in one study, published in three records,^38^^,^^39^^,^^62^ and self-report scales were used in 32 studies, published in 36 records.^27^^,^^28^^,^^30^, ^31^, ^32^^,^^34^^,^^47^, ^48^, ^49^, ^50^, ^51^, ^52^, ^53^, ^54^, ^55^, ^56^, ^57^, ^58^, ^59^, ^60^, ^61^^,^^63^, ^64^, ^65^, ^66^, ^67^^,^^69^, ^70^, ^71^, ^72^, ^73^, ^74^, ^75^, ^76^, ^77^, ^78^ Seven different self-report scales were used, most commonly the Perinatal Post-traumatic Stress Disorder Questionnaire (PPQ). Cut-off points varied across studies, even when the same measure was used. *PTS Prevalence up to one month after birth*

**Table 5:** PTS prevalence data by time of assessment and assessment tool.

**Study ID**	**Assessment tool & cut-off**	**Assessment time**	**Gestational age (weeks)**	**Participants**	**N**	**n**	**%**
**Time of assessment ≤1 month**							
**Clinical diagnosis**							
Misund 2013, 2014, 2016^a^	Clinical review of self-report measures	4–30 days after birth (median=11 days)	<33	Mothers	29	15	52.0
Naeem 2019	Clinician Administered post traumatic-stress disorders scale	3-5 days after birth	24-36	*Parents*	*158*	*38*	*24.0*
				Mothers	79	34	43.0
				Fathers	79	4	5.0
**Impact of Event Scale Revised (IES-R)**							
Yaman 2015	IES-R >30	During admission (≥7 days)	24-37	*Parents*	*132*	*98*	*74.2*
				Mothers	66	54	82.0
				Fathers	66	44	66.7
Aftyka 2014	IES-R >33 (from author)	During admission (mothers: mean 8.0 days SD=3.0; fathers: mean 8.1 days SD=3.3)	Mean 34.3 (SD 4.8)	*Parents*	*66*	*29*	*44.0*
				Mothers	39	20	51.0
				Fathers	27	9	33.0
Goutaudier 2011	IES-R >36	≤3 weeks after birth	27-37	Mothers	27	21	78.0
**Impact of Event Scale (IES)**							
Misund 2013^b^	IES≥19	2 weeks after birth (median=11 days, range=4–30 days)	<33	Mothers	29	4	14.0
		2 weeks after admission	<33	Mothers	27	8	30.0
Misund 2016^b^	IES>19	2 weeks after birth	<33	Mothers	29	13	44.8
**Perinatal Posttraumatic Stress Disorder Questionnaires (PPQ)**							
Holditch-Davis 2009	PPQ≥6	During admission	Mean 27.2 (SD 2.9)	Mothers	177	76	42.9
Holditch-Davis 2015^a^	PPQ≥6	During admission	Mean 27.2 (SD 2.9)	Mothers	232	93	40.1
Garfield 2015^a^	PPQ≥6	3 months after birth (60% during one month) after birth	<37	Mothers	113	*34*	30.0
Koliouli 2016	PPQ>6	During admission	<35	Fathers	48	31	65.8
Vanderbilt 2009	PPQ≥6	Mean=2.5 days after birth (SD=1.7)	Mean 34 (SD 3.8)	Mothers	59	14	24.0
Toly 2019	PPQ≥19	During admission	23-39.3	Mothers	19	7	36.8
Naeem 2019^b^	PPQ>19	1 month after birth	24-36	Mothers	79	38	48.0
**Modified PPQ (mPPQ)**							
Eutrope 2014 a	mPPQ≥19	After birth and before discharge	<32	Mothers	88	31	35.0
Greene 2015 & 2018 a	mPPQ>19	1 month after birth (mean=28.1 days)	23.2-32.3	Mothers	69	17	26.0
**Stanford Acute Stress Reaction Questionnaire (SASRQ)**							
Barr 2010	SASRQ>37 (data from authors)	1 month after admission	≥34	*Parents*	*204*	*59*	*28.0*
				Mothers	110	36	33.0
				Fathers	94	22	23.0
Jubinville 2012	SASRQ>37	7-10 days after birth	<33	Mothers	40	11	28.0
Shaw 2006	SASRQ>38	2-4 weeks after admission	26-41	*Parents*	*40*	*11*	*28.0*
				Mothers	25	11	44.0
				Fathers	15	0	0.0
Shaw 2014	SASRQ ≥38	1 week after birth	26-34	Mothers	135	96	71.1
**PTSD checklist (PCL)**							
Naeem 2019^b^	PCL ≥30	One month after birth	24-36	Fathers	79	28	35.4
Vinall 2018	PCL>33	Before discharge	<37	Mothers	36	2	6.0
**Acute Stress Disorder Scale (ASDS)**							
Lefkowitz 2010	ASDS scoring ≥1 symptom in each category: dissociation, re-experiencing, avoidance & arousal	3-5 days after admission	<30	*Parents*	*128*	*40*	*31.3*
				Mothers	87	30	34.9
				Fathers	41	10	24.4
**Time of assessment >1 month to 1 year**							
**Clinical diagnosis**							
Helle 2018^a^	Structured Clinical Interview for Diagnostic and Statistical Manual of Mental Disorders (SCID)	4-6 weeks after birth	<37	*Parents*	*189*	*21*	*11.0*
				Mothers	111	*17*	14.9
				Fathers	78	*4*	4.8
Misund 2013	Clinical review of self-report measures	7.6–10.4 months after birth (median=8.5 months)	<33	Mothers	29	10	33.0
**Davidson Trauma Scale (DTS)**							
Rodriguez 2020	DTS	7-12 months after birth	<32	Mothers	61	23	38.0
**IES-R**							
Chang 2016	IES-R≥24	6-8 months after birth	<37	Mothers	102	26	15.5
Aftyka 2017	IES-R>33 (from author)	3-12 months after birth	Mean 34.33 (SD 4.8)	*Parents*	*125*	*68*	*43.0*
				Mothers	72	43	60.0
				Fathers	53	25	47.0
Aftyka 2020^b^	IES-R>33	3-12 months after birth	Mean 34.5 (SD 5.10)	*Parents*	*82*	*62*	*75.6*
				Mothers	41	*34*	82.9
				Fathers	41	28	68.5
**IES**							
Misund 2016^b^	IES>19	6 months after birth	<33	Mothers	27	8	30.0
**mPPQ**							
Greene 2015 &2018	mPPQ>19	4 months corrected age	23.2-32.30	Mothers	52	3	6.0
Harris 2018^a^	mPPQ>19	Mean 85.1 ± 40.8 days after birth,	≤32	Mothers	37	3	8.0
**SASRQ**							
Shaw 2009	SASRQ>38	4 months after birth	27-41	*Parents*	*17*	*10*	*58.0*
				Mothers	11	6	55.0
				Fathers	6	4	67.0
**PCL**							
Pace 2020 a	PCL≥30	Term equivalent age (TEA)	<30	*Parents*	*164*	*58*	*35.0*
				Mothers	89	32	36.0
				Fathers	75	26	35.0
		12 months corrected age	<30	Parents	106	25	24.0
				Mothers	55	12	22.0
				Fathers	51	13	25.0
Schecter 2020	PCL>30	1 year after admission	<28-<36	Parents	80	12	15.0
Sharp 2021	PCL-5>33	1 -4 months after birth	< 37 - 41	Mothers	77	18	23.4
Lotterman 2019	PCL≥38	During NICU admission (mean=34.8 days SD=27.3)	32-37	Mothers	91	14	15.8
		6 months after first assessment		Mothers	36	*2*	6.0
Lefkowitz 2010	PCL≥1 re-experiencing symptom, ≥2 avoidance symptoms & ≥3 arousal symptoms	>30 days after T1 (median =32.5 days)	<30	*Parents*	*85*	*11*	*13.0*
				Mothers	60	9	15.0
				Fathers	25	2	8.0
**PPQ**							
Feeley 2011	PPQ>6	6 months (corrected for prematurity)	<32	Mothers	21	5	23.8
Malin 2020	PPQ≥19	3 months after discharge	23-<37	Parents	164	41	25.0
**Time of assessment >1 year**							
**Clinical diagnosis**							
Misund 2013 & 2016	Clinical review of self-report measures	19.2-23.4 months after birth (median=20.6 months)	<33	Mothers	29	7	23.0
**IES-R**							
Ahlund 2009	IES-R>33	2-3 years after birth	24-34	Mothers	24	4	17.0
Clark 2021	IES-R≥33	3 months-5 years after infant death (Mean=38.65 months SD=16.9)	NR	*Parents*	*67*	*10*	*15.0*
				Mothers	40	7	18.0
				Fathers	27	3	11.0
**PPQ**							
Gateau 2021	PPQ≥6	≤24 months corrected age	<37	Mothers	139	46	33.0
Forcada-Guex 2011	PPQ≥6	18 months corrected age	Mean 31 (SD 2)	Mothers	47	16	34.0
Pierrehumb 2003	PPQ≥6	18 months corrected age (mean=18.3 months SD=0.6)	<33	Mothers	50	17	34.0
Gateau 2021	PPQ≥6	≤24 months corrected age	<37	Mothers	139	46	33.0
Zerach 2015	PPQ>19	4-16 years after birth	Mean 25.5 (SD 0.71)	Mothers	78	20	25.6
**PCL**							
Pace 2020	PCL≥30	24 months corrected age	<30	Parents	166	31	18.7
				Mothers	92	17	18.0
				Fathers	74	14	19.0
Sharp 2021	PCL-5>33	1-4 months after birth	< 37-41	Mothers	77	18	23.4
**DTS**							
Rodriguez 2020	DTS	12-<36 months after birth	<32	Mothers	85	41	48.0

Abbreviations: N: Total sample; n: Number of cases.

a Studies measured both anxiety and PTS.

b Not included in meta-analyses; Italics: Calculated data.

**Figure 3:**
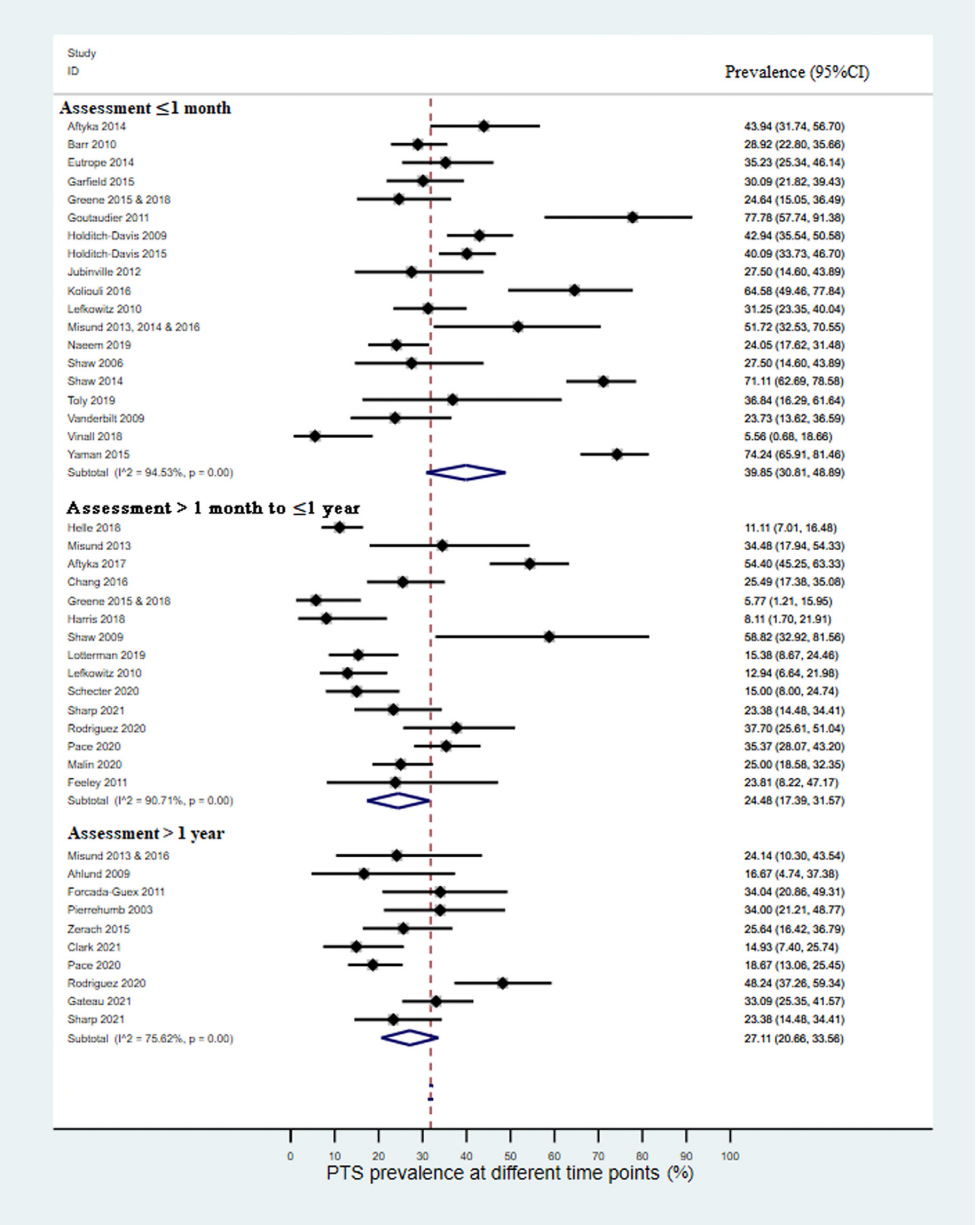
Forest plots of PTS prevalence (%, 95% CI) among parents of babies admitted to NNU assessed ≤ 1 month, > 1 month to ≤ 1 year and > 1 year after birth. Subtotal is the pooled prevalence at each assessment time point; 95% CI is the 95% Confidence Intervals for the PTS prevalence; I^2 represents the quantity of heterogeneity (0-100%); p is the p-value of the heterogeneity test.

The pooled prevalence of PTS across all of the included studies is shown in Table 6 and in forest plots in Figure 3 and Appendix F. The estimated pooled prevalence of PTS up to one month after birth was 39.9% (95%CI: 30.8, 48.9) across 19 studies including 1,800 participants. Individual study estimates ranged from 5.6-74.2% and there was high between-study heterogeneity (I^2^=94.5, p = 0.00). Sub-group analyses showed prevalence of PTS varied when assessed by clinical interviews 24.1% (95%CI: 17.6, 31.5) compared to self-report scales 40.0% (95%CI: 30.8, 49.2) (Table 6). Univariate meta-regression analyses found no evidence of between-study heterogeneity for any variables.

**Table 6:** Pooled prevalence and subgroup analyses of PTS.

**Outcome measure**	**Study (N)**	**Sample (N)**	**Prevalence(95%CI)**	**I^2^%**
PTS≤1 month	19	1,800	39.9 (30.8, 48.9)	94.5
Study setting				
High-income countries	17	1,533	38.6 (30.0, 47.3)	92.7
Middle-income countries	2	267	46.3 (41.4, 51.3)	NA
Study design				
Cohort	15	1,354	35.3 (27.6, 42.9)	89.6
Cross-sectional	4	446	55.0 (33.3, 76.8)	96.1
Sample bias - representativeness				
Representative	6	900	41.1 (26.8, 55.5)	94.4
Non-representative	13	900	39.3 (27.1, 51.6)	95.4
PTS symptoms assessment *^a^				
Clinical interviews	1	158	24.1 (17.6, 31.5)	NA
Self-reported	17	1,613	40.0 (30.8, 49.2)	93.9
Parents				
Mothers	18	1,430	41.7 (32.0, 51.4)	93.9
Fathers	7	370	36.0 (14.0, 58.0)	96.6
Prematurity				
Gestational age (GA)>33 weeks	12	1,037	42.2 (28.0, 56.5)	96.5
GA ≤33	7	763	35.8 (30.0, 41.6)	61.3
Measuring scales ^a^				
PPQ	6	600	39.4 (30.1, 48.6)	81.4
Other scales	12	1,171	38.9 (26.2, 51.5)	95.7
> 1 month ≤ 1 years	15	1,067	24.5 (17.4, 31.6)	90.7%
Study setting				
High-income countries	13	915	24.3 (16.5, 32.2)	91.5
Middle-income countries	2	152	21.4 (15.1, 27.8)	NR
Study design				
Cohort study design	9	433	22.1 (13.6, 30.6)	87.3
Cross-sectional study design	6	634	27.6 (14.2, 40.9)	94.2
Selection bias- representativeness*				
Representative	3	227	11.2 (6.5, 15.9)	NR
Non-representative	12	840	27.3 (19.0, 35.7)	91.0
PTS symptoms assessment* ^a^				
Clinical interviews	1	189	11.1 (7.0, 16.5)	NA
Self-reported	13	851	25.1 (17.2, 33.1)	90.8
Parents ^b^				
Mothers	13	830	25.7 (17.6, 33.8)	88.7
Fathers	5	237	28.5 (9.6,47.4)	93.2
Scales ^a^				
PPQ	2	185	24.9 (18.9, 31.1)	NR
Other scales	12	855	23.9 (15.7, 32.2)	92.4%
PTS > 1 year	10	762	27.1 (20.7, 33.6)	75.6%
Study setting*				
High-income countries	9	677	24.5 (19.5, 29.6)	55.5%
Middle-income countries	1	85	48.2 (37.3, 59.3)	NA
Study design				
Cohort study design	5	370	25.8 (19.2, 32.4)	46.8
Cross-sectional study design	5	392	27.5 (15.9, 39.0)	85.6
Sample representativeness Non-representative	10			
PTS symptoms assessment				
Self-reported	10			
Parents*				
Mothers	10	661	27.6 (21.4, 33.9)	69.2
Fathers	2	101	16.1 (9.0, 23.2)	NR
Prematurity^c^				
GA >33 weeks	3	240	25.68(16.7, 35.0)	45.6
GA ≤33 weeks	6	455	30.5 (20.8, 40.3)	80.2
				
Measuring scales^a^				
PPQ	4	314	31.3 (26.2, 36.4)	0.0
Other scales	5	419	24.3 (13.4, 35.2)	85.6

Abbreviations: ^a^ Misund not included; ^b^ Schecter 2020 and Malin not included as parents data reported combined; ^c^ Clark not included as GA NR;* P <0.05 significant difference between subgroups; NR: Not reported as a few studies were pooled; NR: Not reported; PPQ: Perinatal Posttraumatic Stress Disorder Questionnaires.

### PTS Prevalence up from one month and up to one year after birth

The estimated pooled prevalence of PTS from one month up to one year after birth was 24.5% (95%CI: 17.4 to 31.6) across 15 studies including 1,067 participants. Individual study estimates ranged from 5.8% – 58.8% and there was high between-study heterogeneity (I^2^=90.7, p=0.00). There was evidence of differential prevalence estimates for representative 11.2% (95%CI: 6.5, 15.9) and non-representative studies 27.3% (95%CI: 19.0, 35.7) and between clinical interviews 11.1 % (95%CI: 7.0, 16.5) and self-reported PTS symptoms 25.1% (95%CI: 17.2, 33.1) (Table 6). *PTS Prevalence more than one year after birth*

The estimated pooled prevalence of PTS more than one year after birth was 27.1% (95% CI:20.7, 33.6) across ten studies including 762 participants. Individual study estimates ranged from 14.9 - 48.2% and between-study heterogeneity was high (I^2^= 75.6%, p=0.00). There was evidence of differential prevalence rates between HICs (24.5%; 95%CI: 19.5, 29.6) and MICs (48.2%; 95%CI: 37.3, 59.3) and mothers (27.6 %; 95%CI: 21.4, 33.9) and fathers (16.1%; 95%CI: 9.0, 23.2) (Table 6). Univariate meta-regression analyses found no evidence of between-study heterogeneity for any variable.

## Discussion

This systematic review and meta-analysis is the first to provide an estimate of the prevalence of anxiety and PTS among parents of babies admitted to NNU. There was compelling evidence of high prevalence of anxiety and PTS among parents of babies admitted to NNU and after discharge. The prevalence of anxiety was highest during the first month after birth, with two in five parents experiencing symptoms. The prevalence decreased to approximately one in four parents over the first year after birth, and one small study reported no anxiety more than one year after birth. These rates are considerably higher than the prevalence of anxiety among women during the perinatal period, which is estimated to be 15-20%,^80^^,^^81^ and among women in the general population, which is estimated to be 5-9%. ^82^ The prevalence of PTS was also highest in the first month after birth, with almost two in five parents experiencing symptoms. The prevalence decreased to just over one in four parents over the first year after birth, but remained high more than one year after birth. Again, this is considerably higher than the prevalence of PTS among women during the perinatal period and among women in the general population, which is estimated to be approximately 4-10%.^83^, ^84^, ^85^ The review findings therefore suggest that both anxiety and PTS are more prevalent and persistent in parents of babies admitted to NNU.

The studies in the review were heterogeneous in their design, setting, inclusion and exclusion criteria, level of NNU and length of stay, characteristics of parents and babies, assessment methods, tools and time points. There was also considerable variation in the prevalence estimates of both anxiety and PTS across the included studies. Evidence from subgroup analyses and meta-regression suggested that some of the variation in prevalence estimates could be explained by study heterogeneity, in particular sex of parent, assessment method and tool used for assessment of anxiety and PTS.

Anxiety was higher in mothers than fathers. Similar disparity has been found in reviews of parental stress related to premature birth^16^^,^^86^ and in the broader perinatal population.^87^ The reason for this disparity is unclear and requires further investigation. Previous research suggests such differences may reflect variations in expectations of the role played by mothers and fathers within the NNU culture^88^ and in society more broadly.^86^ A focused ethnography study in the UK found that fathers engaged in considerable effort to manage their emotions as they attempted to reconcile the tension between what they felt and what they thought others expected them to feel.^89^ PTS was high for both mothers and fathers and, although the prevalence differed significantly more than a year after birth, this disparity should be considered with caution due to the small number of studies in the analysis.

Prevalence also varied by how anxiety and PTS were measured, with lower prevalence rates when structured clinical interviews were used. This is consistent with other reviews on anxiety in general perinatal populations,^80^ yet contrasts with a systematic review of postpartum PTS which found no difference in prevalence based on clinical interviews or self-report measures.^90^ As with this review, the number of studies using clinical interviews to assess anxiety and PTS was very low. While clinical interviews are considered the gold standard for diagnosing mental disorders, it is not feasible to conduct a clinical interview with all parents of babies in NNU. The STAI and PPQ were the most common self-report assessment tools used to measure anxiety and PTS, respectively. Variation was found according to the specific tool used to assess anxiety with higher prevalence rates based on the STAI compared to other self-report measures. More research is needed comparing the STAI to clinical interviews to explore the validity of this higher prevalence before considering its use in a clinical environment.

The strengths of this review include a broad and comprehensive search strategy across multiple databases with no language or date restrictions and a thorough grey literature search. The approach was inclusive and robust with screening, data extraction and analysis all performed and cross-checked by at least two independent reviewers. A particular strength of the review is the inclusion of parents and primary caregivers of all NNU babies. In addition, the review estimates the prevalence of anxiety and PTS across three different time-points, providing insight into the prevalence of these mental health conditions in parents of NNU babies during and beyond the first year after birth. A further strength is the extensive and diverse subgroup analysis and meta-regression, which identified high heterogeneity across studies. However, the heterogeneity imposed a limitation on the ability of the review to provide an precise estimation of anxiety and PTS prevalence. In addition, specific groups of parents, notably those parents of infants with congenital anomalies, parents whose babies had died and parents with pre-existing mental health conditions were frequently excluded from studies. There were also limited data on key social determinants of health, for example, parents experiencing social deprivation and ethnicity of parent. As these groups of parents are at high risk of anxiety and PTS, the pooled prevalence rates for PTS and anxiety in the current review may be an underestimate of the true prevalence for all parents of babies admitted to NNU. A further limitation was the omission of three studies,^91^, ^92^, ^93^ the full texts of which could not be obtained despite extensive searching.

While we found no difference in prevalence of anxiety or PTS in cohort studies in comparison to studies using cross-sectional designs, it is widely recognised that the use of cross-sectional study designs or convenience samples are less likely to lead to representative results. Therefore further large-scale population-based, prospective cohort studies are required to investigate prevalence of mental health conditions in parents of babies admitted to NNU. Future studies should include full demographic details of participants and employ robust standardised measures with validated cut-off points that are sensitive and reliable for this population, using core outcome sets where these are available. The number of participants approached, consented and included in the analysis should be clearly reported to enable a statistical comparison between those who participated and those who did not. Analyses plans should include models that control for confounding variables that may be related to anxiety or PTS symptoms in parents whose babies are admitted to NNU. In addition, research on outcome measures is recommended. In particular, studies comparing STAI against clinical interviews is needed to better understand the higher prevalence levels obtained using STAI and ultimately to avoid significant numbers of false positive cases and subsequent additional demands on resources in practice. Future studies of prevalence of common mental disorders should be inclusive of the whole NNU parent population and be large enough to explore subgroup variability. Further research is also needed on social determinants of health which may impact on mental health, including groups which are underrepresented in existing studies, for example, fathers and other carers, parents and carers from black and minority ethnic backgrounds, and parents and carers from low and middle income countries.

The current findings have significant implications for practice and highlight the necessity for routine mental health screening for parents of babies admitted to NNU as part of standard care in the year after birth. A recent systematic review^94^ suggests that universal screening for parent mood and anxiety disorders in NNUs may be feasible with a tool that is brief enough to use in clinical settings.^95^ Alternatively, a series of short screening questions may be used to identify parents who may benefit from intensive assessment. For example, the Whooley questions (two questions for depression)^96^ and GAD2 (two questions for anxiety)^97^ are routinely used as a first step in identifying women with perinatal mental health problems in the UK.^98^ In addition to identifying parents with mental health problems, preventative and supportive interventions for parents in NNU are needed. Priority should be given to implementing evidence based interventions that are effective in decreasing parental anxiety and trauma symptoms in the NNU in policy and practice.Consideration should be given to making mental health support part of routine care for specific groups, for example, parents of very preterm infants with extended stays.

The review indicated a high prevalence of anxiety and PTS among the parents of babies admitted to NNU, which persists long after discharge. Meta-analyses showed that anxiety and PTS affect two in five parents to babies admitted to NNU, and these are higher than rates in the general perinatal population. However high statistical heterogeneity across included studies and the exclusion of some high-risk subgroups suggest that the pooled prevalence rate should be interpreted with caution. Nevertheless, rates of anxiety and PTS were consistently higher than the general perinatal population highlighting the need for routine screening and a clearer pathway to mental health intervention for parents of babies admitted to NNU. A routine and standardised screening strategy which includes, at the very least, assessment during NNU admission and after discharge in the year after birth is recommended. Timely recognition of anxiety or PTS symptoms by health professionals is paramount. Adequate health service resources should be in place to ensure early referral and appropriate interventions are offered.

## Contributors

RM designed the review along with FA and SH, completed the literatures search, screened abstracts and full text and assessed studies for eligibility for inclusion at all stages of the review process, did data extraction, did the analysis, drafted the initial manuscript, and reviewed and revised the manuscript. SH designed the review along with RM and FA, screened abstracts and full text and assessed studies for eligibility for inclusion at all stages of the review process, did data extraction, reviewed the analysis, drafted the initial manuscript along with RM and FA and reviewed and revised the manuscript. HB contributed to the design of the review, did abstract and full text screening, data extraction and reviewed and revised the manuscript. CG, LF and AS contributed to the design of the review, assisted with design of the data extraction form, reviewed the extracted data and revised the manuscript. FA conceptualised and designed the review, contributed to data screening and data extraction, supervised initial analysis of data, drafted the initial manuscript with RM and SH, and reviewed and revised the manuscript. All authors approved the final manuscript as submitted and agree to be accountable for all aspects of the work.

## Funding

This research is funded by the National Institute for Health Research (NIHR) Policy Research Programme, conducted through the Policy Research Unit in Maternal and Neonatal Health and Care, PR-PRU-1217-21202. The views expressed are those of the author(s) and not necessarily those of the NIHR or the Department of Health and Social Care.

## Declaration of interests

NIHR funded the project and at least part of the salary of FA, SH, RM through the Policy Research Unit in Maternal and Neonatal Health and Care. CG and AS are part of the NIHR Policy Research Unit in Maternal and Neonatal Health and Care but are not paid by the NIHR.CG is supported by the Medical Research Council through a Clinician Scientist Fellowship and this supported his salary over the time spent on this review. All other authors have none to declare.

## Appendix Supplementary materials

Image, application 1

Image, application 2
