# Supplementary material for: Prevalence of anxiety and post-traumatic stress (PTS) among the parents of babies admitted to neonatal units: A systematic review and meta-analysis
Source: eClinicalMedicine. 2021 Dec 21;43:101233. doi: 10.1016/j.eclinm.2021.101233 (PMC8713115; doi:10.1016/j.eclinm.2021.101233)

**Appendix A: Database: Medline (Ovid MEDLINE® Epub Ahead of Print, In-Process & Other Non-Indexed Citations, Ovid MEDLINE® Daily and Ovid MEDLINE®) 1946 to 24/January/2020**

**Search Strategy:**

| Searches | Results |
| --- | --- |
| Stress Disorders, Post-Traumatic/ | 31541 |
| Anxiety/ or exp Anxiety Disorders/ | 147646 |
| Stress, Psychological/ | 117413 |
| sleep disorders, intrinsic/ or "sleep initiation and maintenance disorders"/ | 12900 |
| Fatigue/ | 27888 |
| (posttraumatic or post-traumatic or ptsd or trauma*).mp. | 419885 |
| (anxiet* or anxious).mp. | 233293 |
| (phobia? or phobic).mp. | 17069 |
| (obsessive compulsive disorder* or ocd).mp. | 19955 |
| (((psycholog* or mental or acute) adj2 stress*) or stressful).mp. or stress*.ti. | 336898 |
| (insomnia or sleepless* or ((problem? or disturbed or disrupt* or difficult*) adj2 sleep*)).mp. | 32845 |
| (fatigue* or tired*).mp. | 108198 |
| ((panic adj2 (attack? or disorder?)) or palpitation?).mp. | 18626 |
| (fear? or worry or worries or concern? or crisis or crises).mp. | 451910 |
| 1 or 2 or 3 or 4 or 5 or 6 or 7 or 8 or 9 or 10 or 11 or 12 or 13 or 14 | 1480981 |
| exp Parents/ | 108989 |
| Caregivers/ | 34915 |
| (parent or parents or father* or mother* or mums or moms or dads or maternal or paternal or carer? or caregiver? or care giver? or caretaker? or care taker? or guardian?).mp. | 798493 |
| 16 or 17 or 18 | 798493 |
| Intensive Care Units, Neonatal/ | 14162 |
| Infant, Newborn/ and (Intensive Care Units/ or Critical Care/) | 3886 |
| exp infant, low birth weight/ or exp infant, premature/ | 78068 |
| ((infan* or neonat* or newborn? or baby or babies or preterm) adj5 (intensive care or intensive therap* or critical care or unit?)).ti,ab. | 27226 |
| ((high dependency or special care) adj5 (infan* or neonat* or newborn? or baby or babies)).mp. | 939 |
| (nicu or scbu).ti,ab. | 9493 |
| ((infan* or neonat* or newborn? or baby or babies) and (prematur* or preterm or low birth weight or "small for gestational age" or vlbw or lbw or sga)).ti. | 39870 |
| 20 or 21 or 22 or 23 or 24 or 25 or 26 | 112387 |
| prevalence/ or incidence/ or epidemiology/ or morbidity/ | 550306 |
| Cross-sectional studies/ | 316346 |
| (prevalence or prevalent).mp. | 797811 |
| (incidence or incident?).mp. | 899309 |
| epidemiolog*.mp. | 1841162 |
| (frequenc* or occurence* or pattern? or burden).mp. | 2372751 |
| ((cross-sectional or crossectional) adj (stud* or survey?)).ti,ab. | 188634 |
| 28 or 29 or 30 or 31 or 32 or 33 or 34 | 4867800 |
| 15 and 19 and 27 and 35 | 1025 |
| exp animals/ not humans/ | 4666511 |
| 36 not 37 | 1022 |

**Appendix B:** Risk of bias assessment tool, adapted from the Risk of Bias Tool for Prevalence Studies developed by Hoy et al

| **Risk of Bias Item** | **Rating: High, Low, Unclear** |
| --- | --- |
| 1. Was the study target population a close representation of the parents to babies admitted to NNU? |  |
| 1. Was the sampling frame a true or close representation of the parents admitted to NNU? |  |
| 1. Was some form of random selection used to select the sample, OR, was a census undertaken? |  |
| 1. Were data collected directly from the parents? |  |
| 1. Was anxiety/PTS well defined? |  |
| 1. Were validated and reliable measures used? |  |
| 1. Was the same method used for data collection? |  |
| 1. Was the length of assessment time appropriate? |  |
| 1. Were the numerator(s) and denominator(s) for the   calculation of the prevalence reported and appropriate? |  |

**Appendix C: Risk of Bias of included studies - anxiety**

| **Study ID** | **Population represents the national population** | **Study sample represents the target population** | **Study sample selection** | **Non- response bias** | **Direct data collection** | **Anxiety well defined** | **Validity/**  **reliability of measures** | **Same method used for data collection** | **Length of assessment time appropriate** | **Prevalence data** |
| --- | --- | --- | --- | --- | --- | --- | --- | --- | --- | --- |
|  | Low:  > 1 centre | Low: Including both parents to NNU babies without applying any restrictions | Low: Random selection/ census was undertaken | Low:  No difference between responders and non-responders or > 75% response rate | Low:  Data directly collected from parents via self-reported or clinical interviews | Low:  Using standard measure | Low:  Using standard measure with a cut-off point | Low:  Same mode of data collection was used for al | Low:  ASD assessed < 1 month; PTSD > 1 month post birth/NNU admission | Low: Numerator& denominator of prevalence reported & appropriate |
| **Bonacquisti, 2020** | Low | High | High | High | Low | Low | Low | Low | Low | High |
| **Cajiao-Nieto 2021** | High | High | High | Unclear | Low | Low | Low | Low | Low | Low |
| **Carter 2007** | High | Low | Low | Low | Low | Low | Low | Low | Low | High |
| **Dantas 2012** | Low | Low | High | High | Low | Low | Low | Low | Low | Low |
| **Das 2021** | High | High | High | Low | Low | Low | Low | Low | Low | Low |
| **Eutrope 2014 ^a^** | Low | High | High | Unclear | Low | Low | Low | Low | Low | High |
| **Garfield 2015 ^a^** | Low | High | High | Unclear | Low | Low | Low | Low | Low | Low |
| **Gonzalez-Hernandez 2019** | High | High | High | Unclear | Low | Low | Low | Low | Low | Low |
| **Greene 2015 & 2018^a^** | High | High | High | High | Low | Low | Low | Low | Low | Low |
| **Harris 2018^a^** | Low | High | High | High | Low | Low | Low | Low | Low | Low |
| **Helle 2016 ^a^** | Low | High | High | High | Low | Low | Low | Low | Low | High |
| **Holditch-Davis 2015 ^a^** | Low | High | Low | Unclear | Low | Low | Low | Low | Low | High |
| **Kong 2013** | High | High | High | Unclear | Low | Low | Low | Low | Low | Low |
| **Misund 2014 & 2016^a^** | High | High | High | Low | Low | Low | Low | Low | Low | High |
| **Mulder 2014** | High | Low | Low | Low | Low | Low | Low | Low | Low | Low |
| **Ong 2019** | High | High | High | Low | Low | Low | Low | Low | Low | Low |
| **Onay 2021** | High | High | High | Unclear | Low | Low | Low | Low | Low | Low |
| **Pace 2016 ^a^** | High | High | High | High | Low | Low | Low | Low | Low | Low |
| **Rogers 2013** | High | High | High | Low | Low | Low | Low | Low | Low | High |
| **Segre 2014 & McCabe-Beane 2018** | High | High | High | High | Low | Low | Low | Low | Low | Low |
| **Trumello 2018** | High | High | High | Unclear | Low | Low | Low | Low | Low | High |

Abbreviations: ^a^ studies included in both anxiety and PTS

**Appendix D: Anxiety prevalence and meta-analyses at different time points**

**Anxiety prevalence≤1 month - study setting**


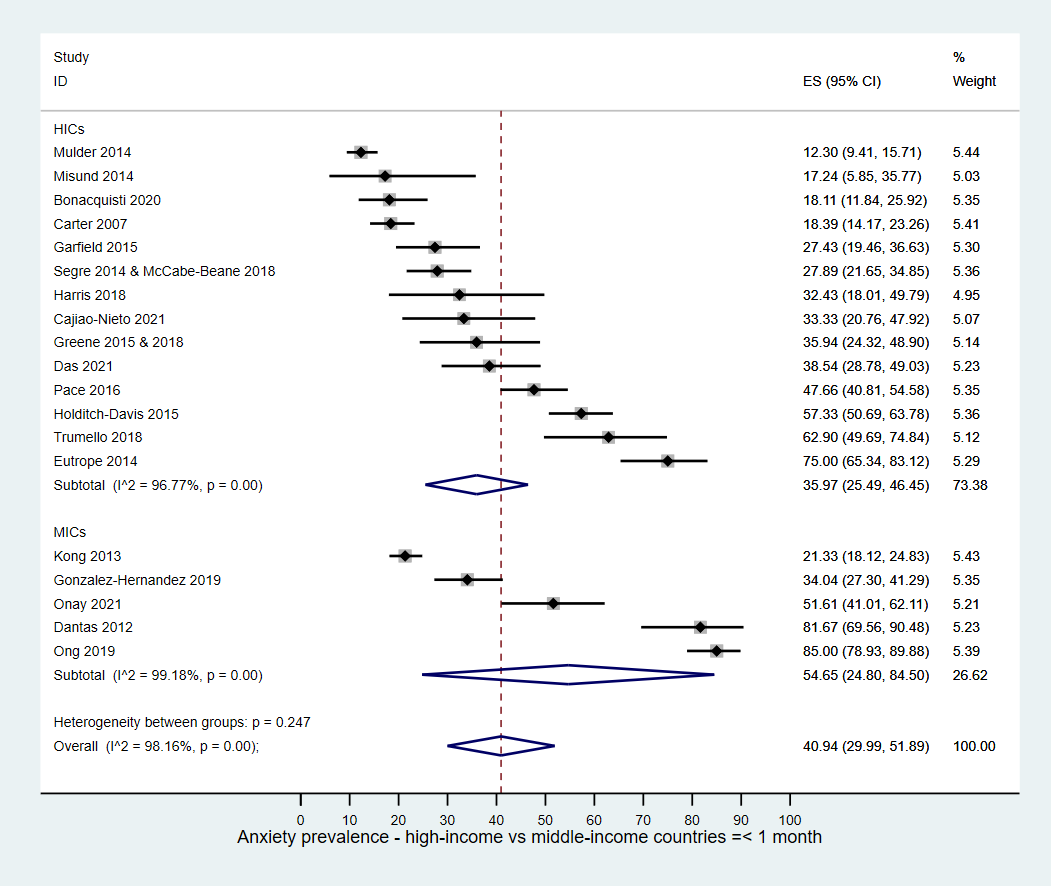


**Anxiety prevalence≤1 month - study design**

**
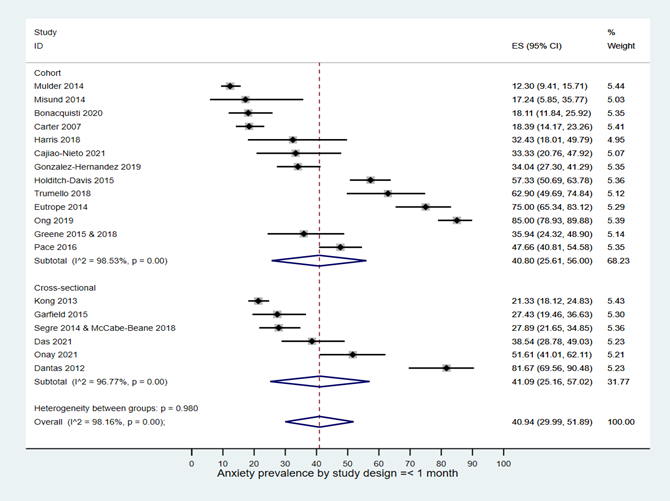
**

**Anxiety prevalence ≤ 1 month-selection bias – sample representativeness**

**
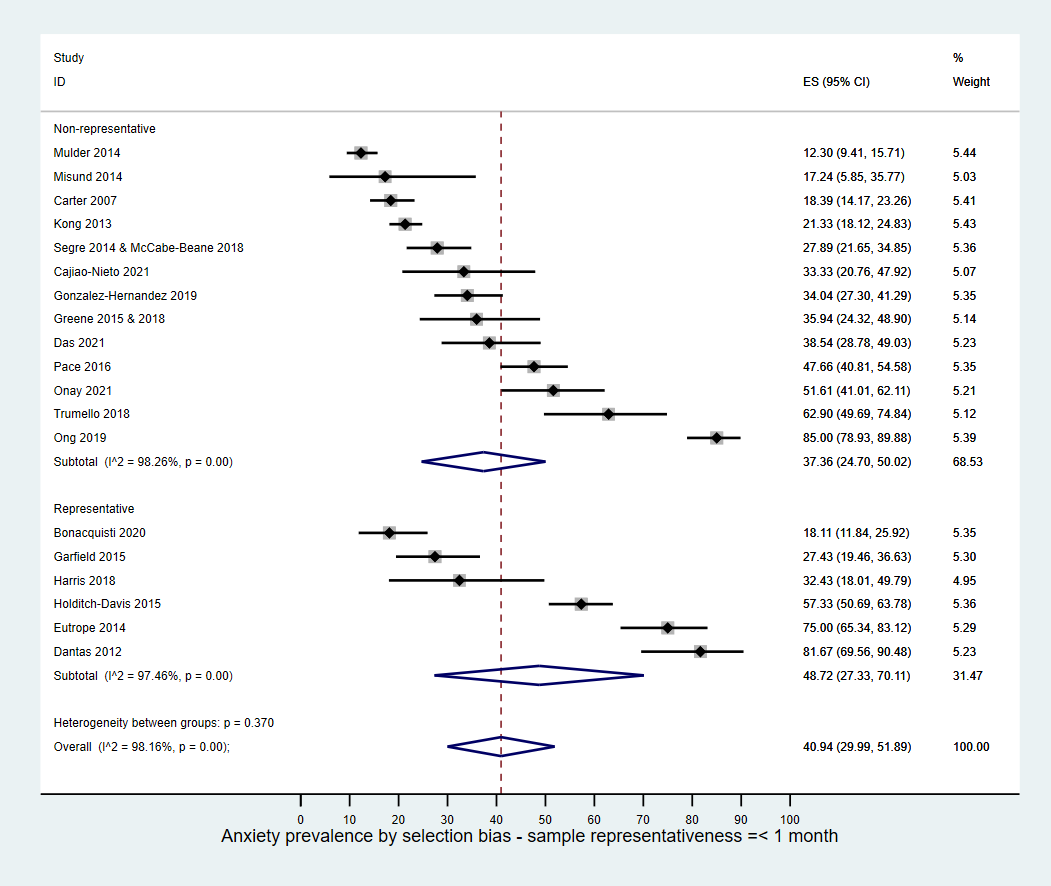
**

**Anxiety prevalence ≤1 month – parents***


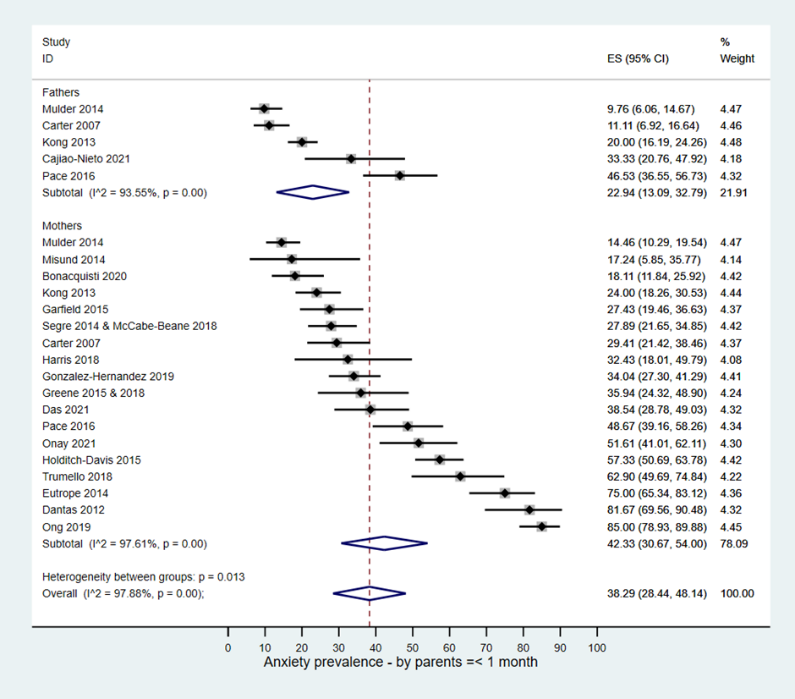


**Anxiety prevalence≤1 month –** **prematurity**

**
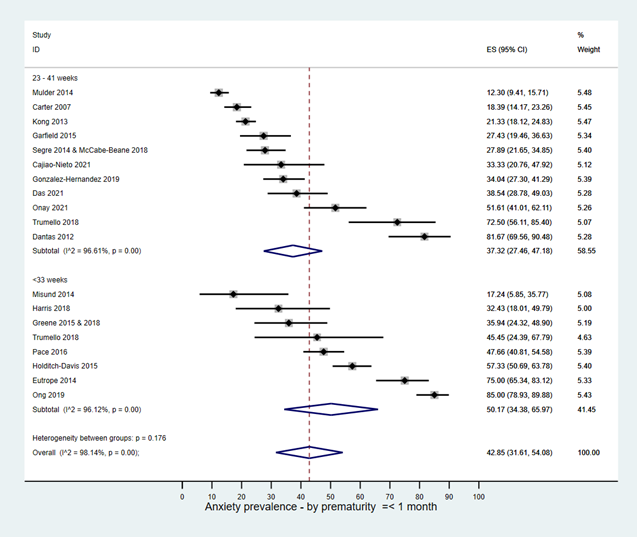
**

**Anxiety prevalence ≤1 month-measuring scales***

**
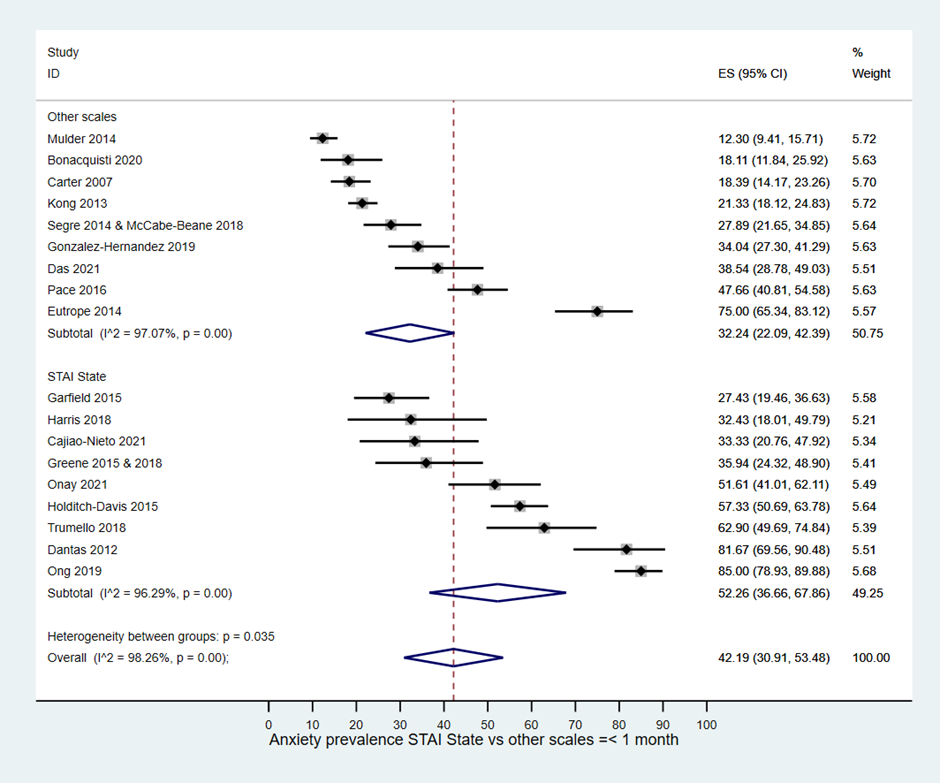
**

**Anxiety prevalence >1 month to ≤ 1 year – study deign**

**
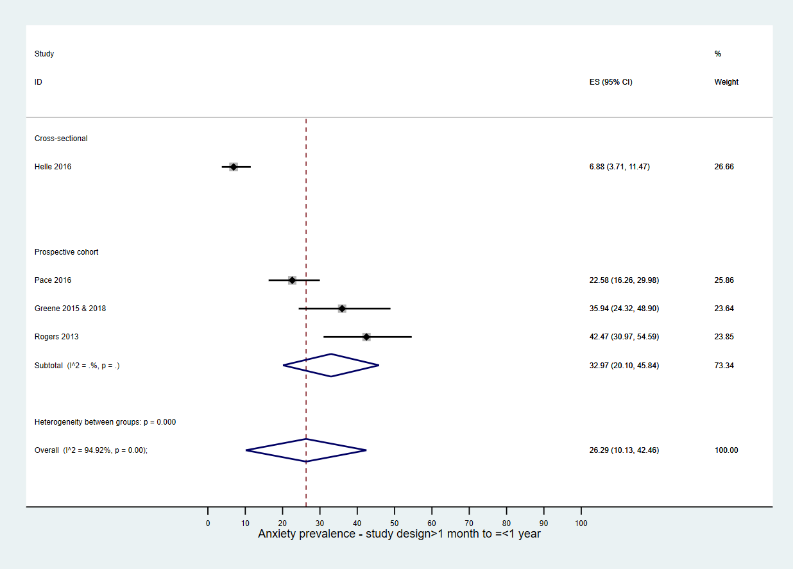
**

**Anxiety >1 month to ≤1year-selection bias - sample representativeness***

**
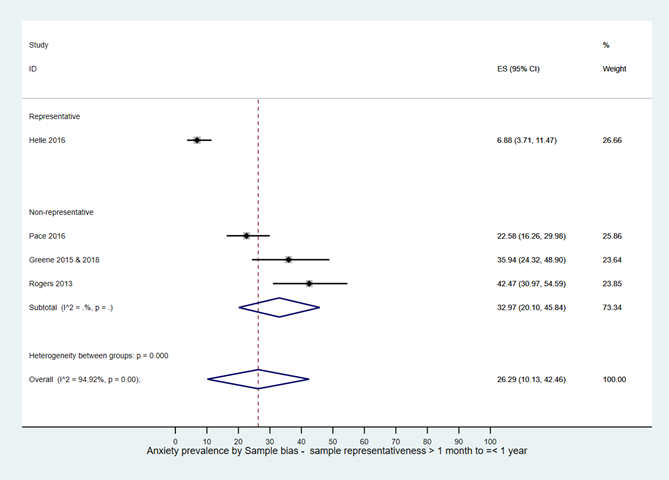
**

**Anxiety >1 month to≤ 1 year-anxiety symptoms***

**
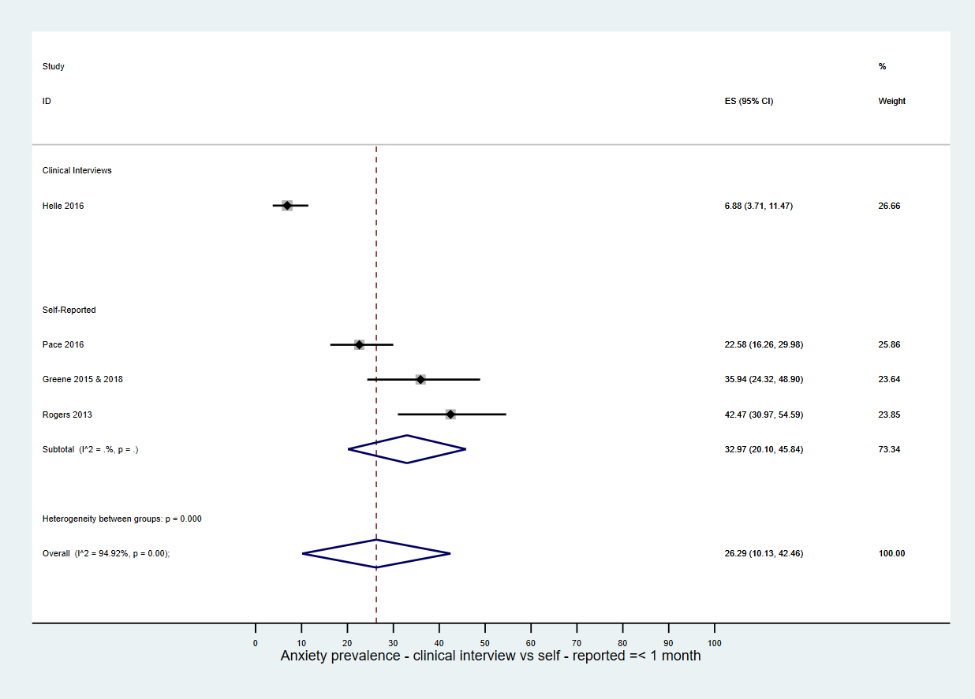
**

**Anxiety prevalence>1 month to ≤1 year-parents***


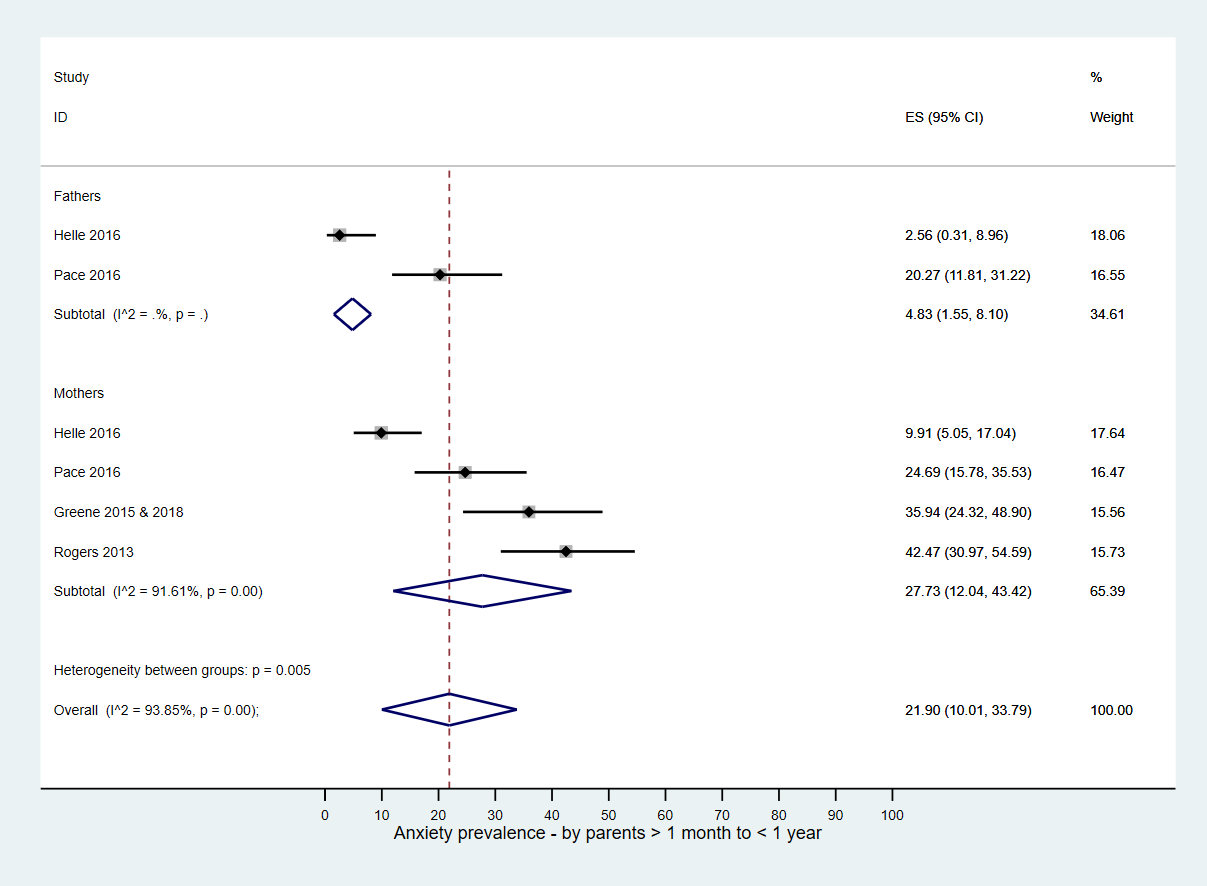


**Anxiety prevalence>1 month to≤1 year- prematurity***

**
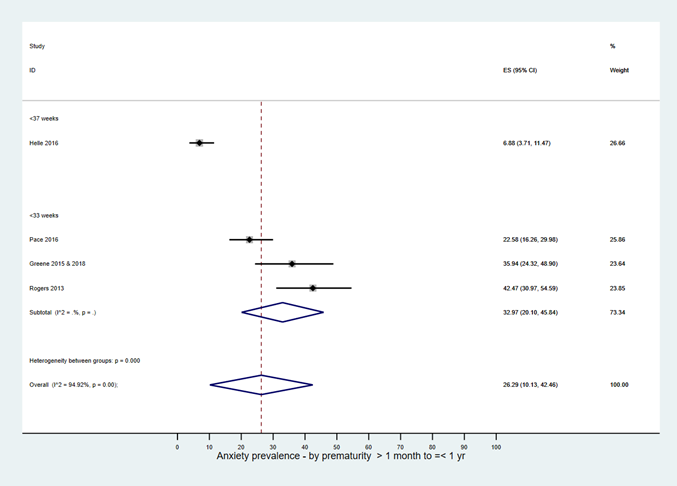
**

**Anxiety prevalence >1 month to≤ 1 year-measuring scales***


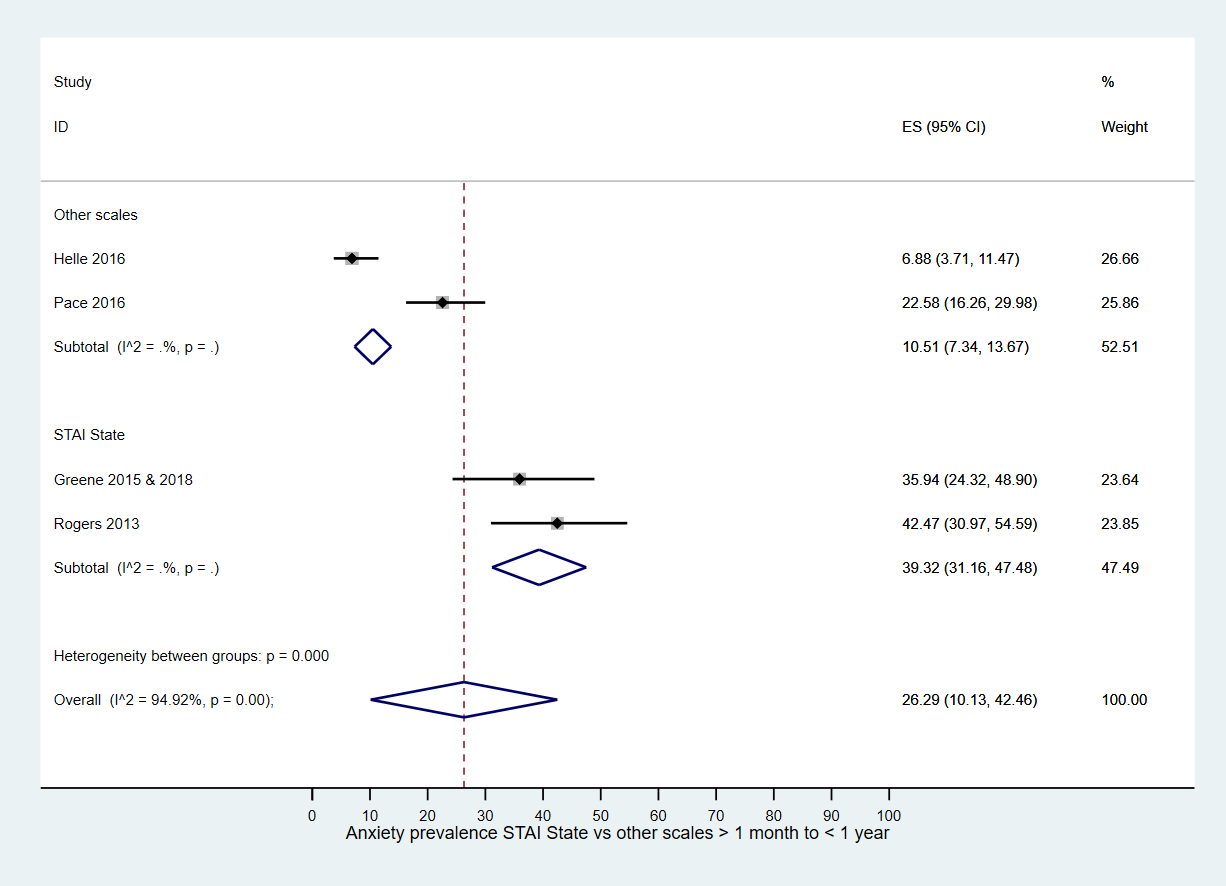


**Appendix E: Risk of Bias of included studies - PTS**

| **Study ID** | **Population representative of the national population** | **Study sample represents the target population** | **Study sample selection** | **Non- response bias** | **Direct data collection** | **Anxiety well defined** | **Validity/**  **reliability of measures** | **Same method used for data collection** | **Length of assessment time appropriate** | **Prevalence data** |
| --- | --- | --- | --- | --- | --- | --- | --- | --- | --- | --- |
|  | Low:  > 1 centre | Low: Including both parents to NNU babies without applying any restrictions | Low: Random selection/ census was undertaken | Low:  No difference between responders and non-responders/ > 75% response rate | Low:  Data directly collected from parents via self-reported or clinical interviews | Low:  Using standard measure | Low:  Using standard measure with a cut-off point | Low:  Same mode of data collection was used for all | Low:  ASD assessed < 1 month; PTSD > 1 month post birth/NNU admission | Low:  Numerator(s) & denominator (s) of prevalence reported & appropriate |
| **Aftyka’s study** | |  |  |  |  |  |  |  |  |  |
| Aftyka 2014 | High | High | High | High | Low | Low | Low | Low | Low | Low |
| Aftyka 2017 & 2020 | High | High | High | High | Low | Low | Low | Low | Low | Low |
| **Ahlund 2009** | High | High | High | Low | Low | Low | Low | Low | High | Low |
| **Barr 2010** | High | High | High | Low | Low | Low | Low | Low | Low | Low |
| **Chang 2016** | High | High | High | Unclear | Low | Low | Low | Low | Low | Low |
| **Clark 2021** | High | High | High | High | Low | Low | Low | Low | Low | Low |
| **Eutrope 2014^a^** | Low | High | High | Unclear | Low | Low | Low | Low | Low | Low |
| **Feeley 2011** | Low | High | High | Unclear | Low | Low | Low | Low | Low | Low |
| **Forcada-Guex 2011** | High | High | High | Low | Low | Low | Low | Low | Low | Low |
| **Garfield 2015^a^** | High | High | High | Unclear | Low | Low | Low | Low | Low | Low |
| **Gateau 2021** | High | High | Unclear | Low | Low | Low | Low | Low | Low | Low |
| **Goutaudier 2014** | High | High | High | High | Low | Low | Low | Low | Low | Low |
| **Greene 2015& 2018^a^** | High | High | High | Unclear | Low | Low | Low | Low | Low | Low |
| **Harris 2018^a^** | Low | High | High | High | Low | Low | Low | Low | Low | Low |
| **Helle 2018 ^a^** | Low | High | High | High | Low | Low | Low | Low | Low | High |
| **Holditch-Davis 2009** | Low | High | Low | Unclear | Low | Low | Low | Low | High | High |
| **Holditch-Davis 2015 ^a^** | Low | High | Low | Unclear | Low | Low | Low | Low | High | High |
| **Jubinville 2012** | High | High | High | Low | Low | Low | Low | Low | Low | Low |
| **Koliouli 2016** | High | High | High | Unclear | Low | Low | Low | Low | Low | Low |
| **Lefkowitz 2010** | High | High | High | Low | Low | Low | Low | Low | Low | Low |
| **Lotterman 2019** | High | High | High | Low | Low | Low | Low | Low | Low | High |
| **Malin 2020** | High | High | High | Low | Low | Low | Low | Low | Low | Low |
| **Misund 2013, 2014 & 2016** | High | High | High | Low | Low | Low | Low | Low | High | High |
| **Naeem 2019** | Low | High | High | Unclear | Low | Low | Low | Low | Low | Low |
| **Pace 2020 ^a^** | High | High | High | Low | Low | Low | Low | Low | Low | Low |
| **Pierrehumber2003** | High | High | High | Low | Low | Low | Low | Low | Low | Low |
| **Rodriguez 2020** | High | High | High | Low | Low | Low | Low | Low | Low | Low |
| **Sharp 2021** | High | High | High | Low | Low | Low | Low | Low | Low | Low |
| **Shaw 2006 & 2009** | High | High | High | High | Low | Low | Low | Low | Low | Low |
| **Shaw 2014** | High | High | High | High | Low | Low | Low | Low | Low | Low |
| **Schecter 2020** | High | Low | High | Unclear | Low | Low | Low | Low | Low | High |
| **Toly 2019** | High | High | High | High | Low | Low | Low | Low | Low | Low |
| **Vanderbilt 2009** | High | High | High | High | Low | Low | Low | Low | Low | Low |
| **Vinall 2018** | High | High | High | Low | Low | Low | Low | Low | Low | Low |
| **Yaman 2015** | Low | Low | High | Low | Low | Low | Low | Low | Low | Low |
| **Zerach 2015** | High | High | Low | High | Low | Low | Low | Low | High | High |

^a^ studies included in both anxiety and PTS

**Appendix F: Post traumatic stress (PTS) prevalence and meta-analyses at different time points**

**PTS prevalence≤1 month-study setting**

**
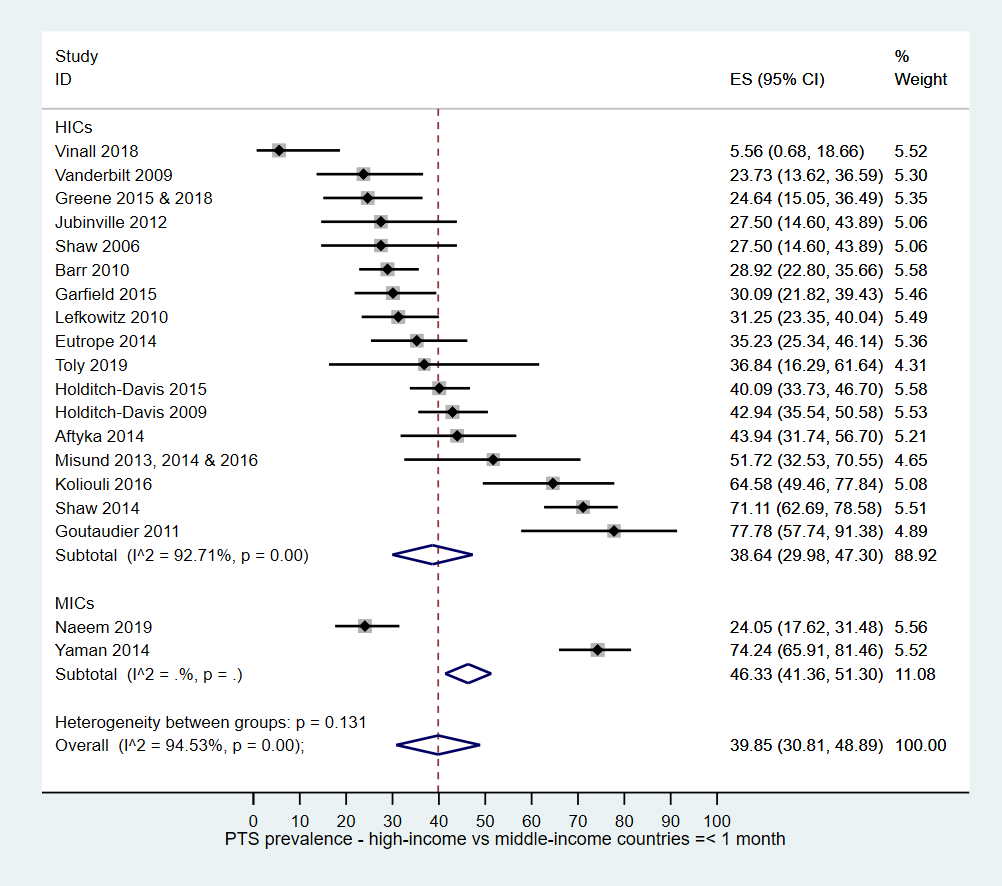
**

**PTS prevalence≤1 month-study design**

**
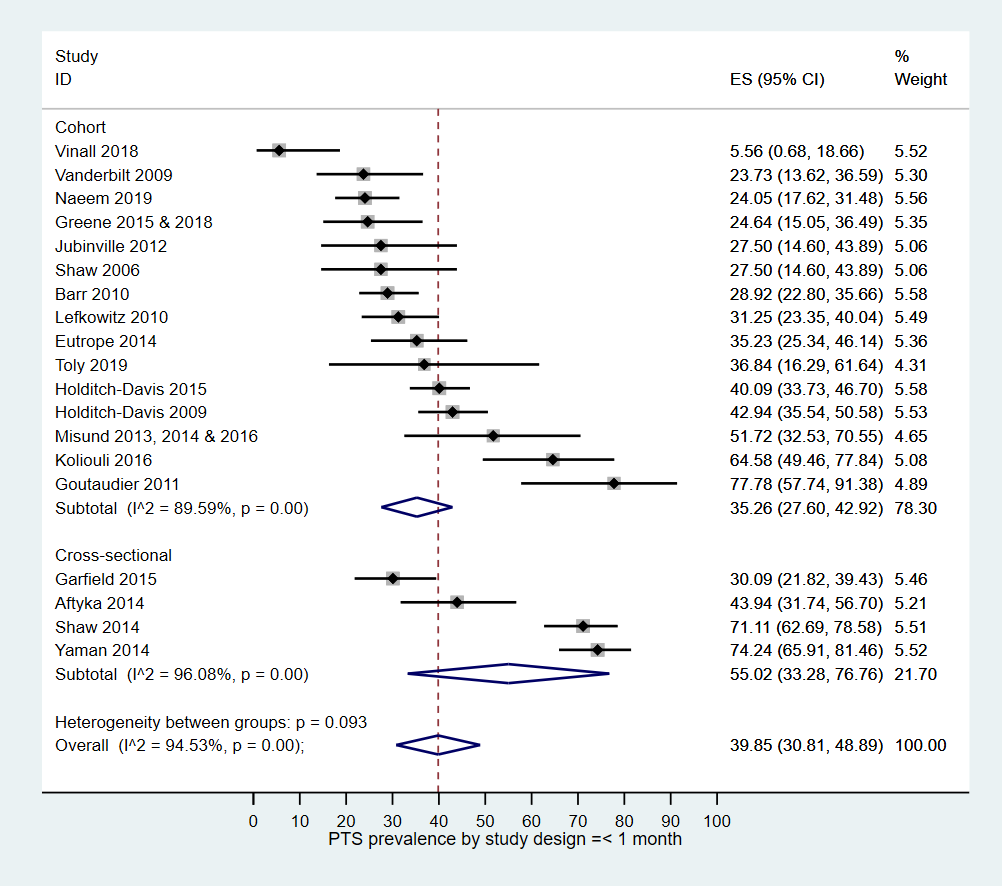
**

**PTS prevalence≤1 month-selection bias-** **representativeness**

**
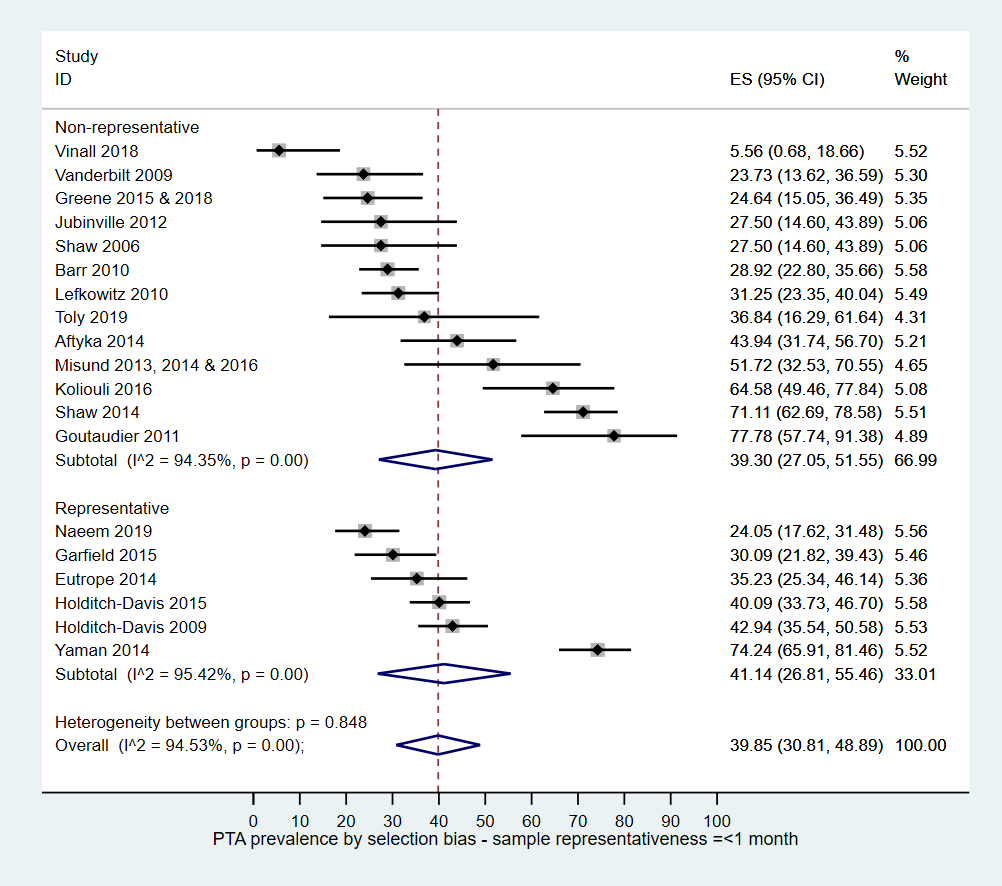
**

**PTS prevalence≤1 month-PTS symptoms***

**
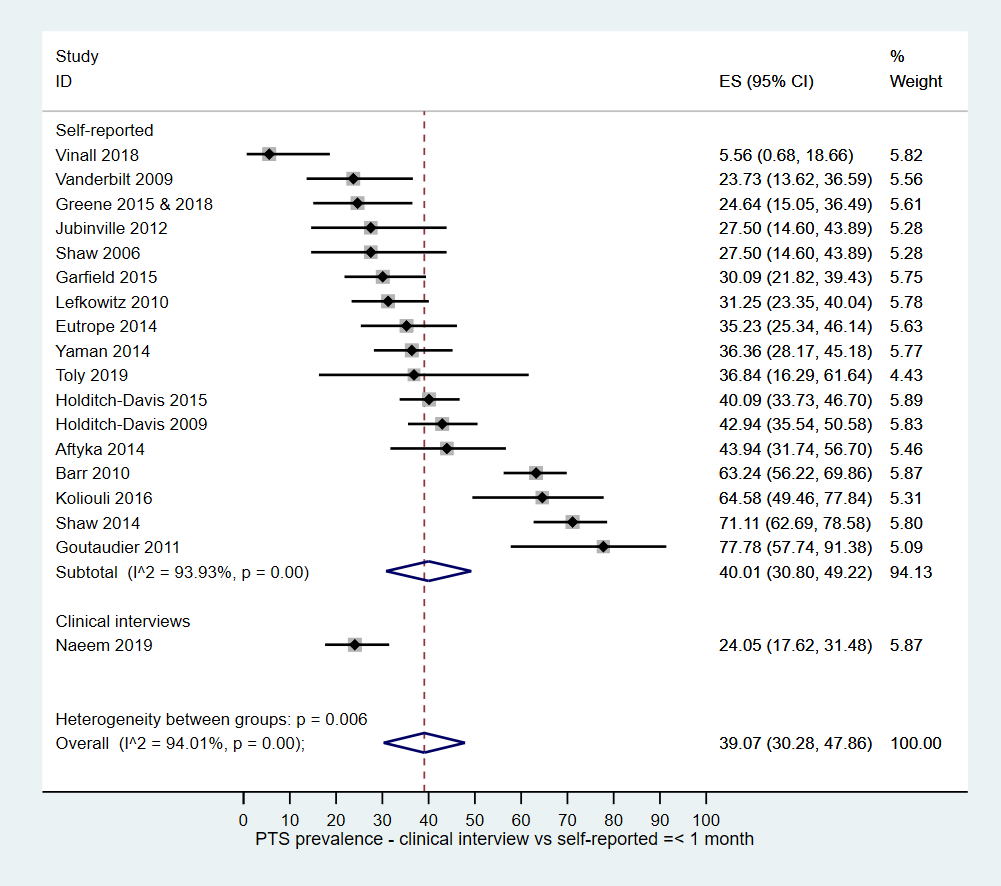
**

**PTS prevalence≤1 month-parents**

**
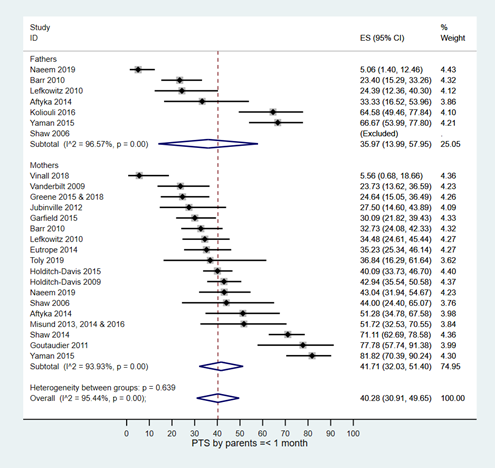
**

**PTS prevalence≤1 month-prematurity**

**
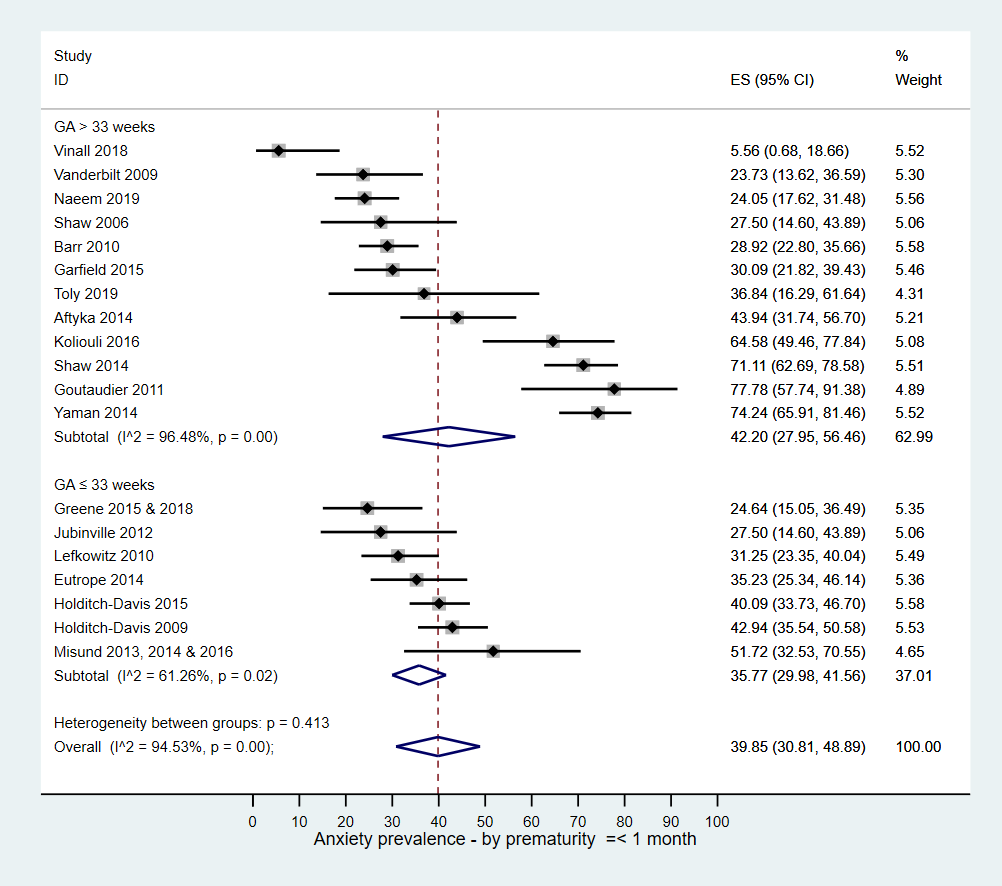
**

**PTS prevalence≤1 month-measuring scales**

**
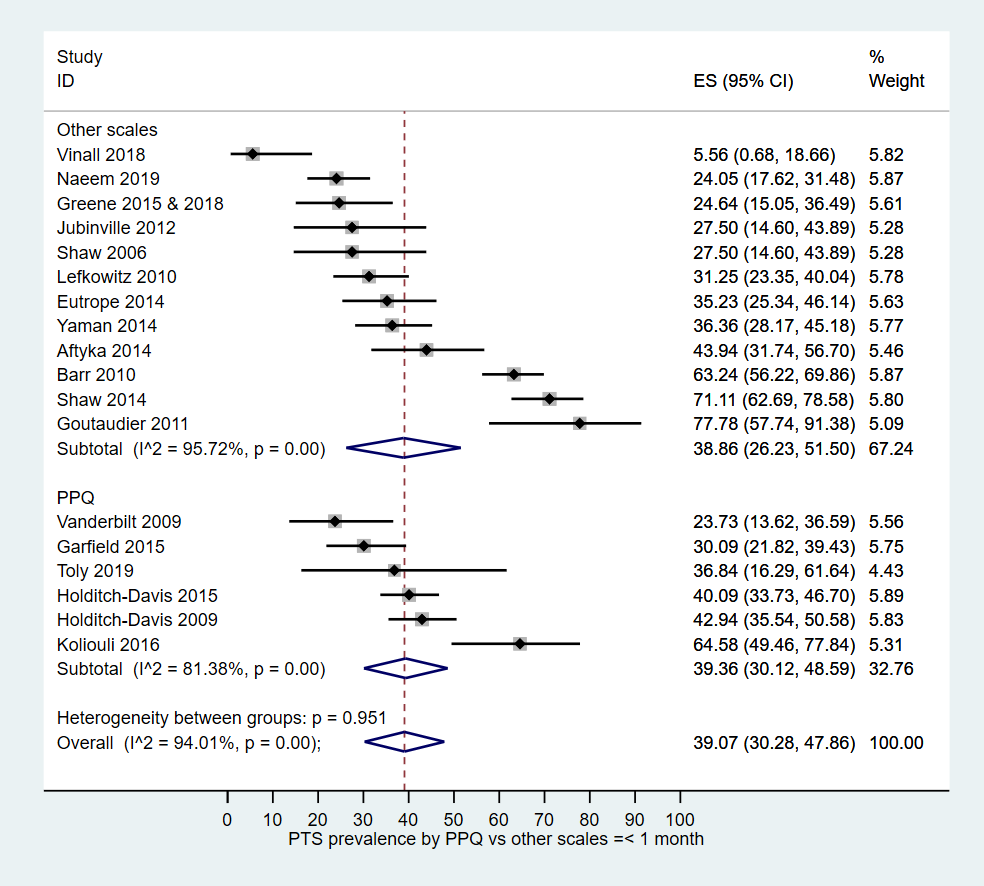
**

**PTS prevalence > 1 month to ≤ 1 year – study setting**

**
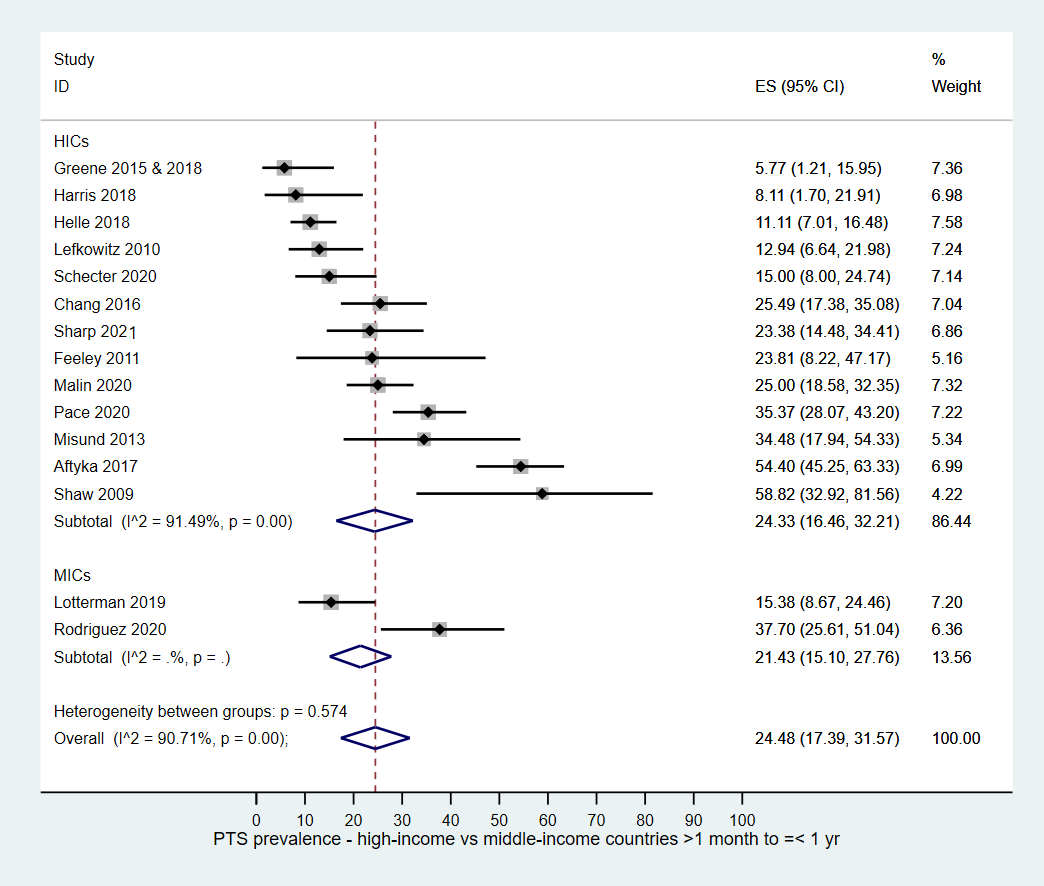
**

**PTS prevalence > 1 month to ≤ 1 year – study design**


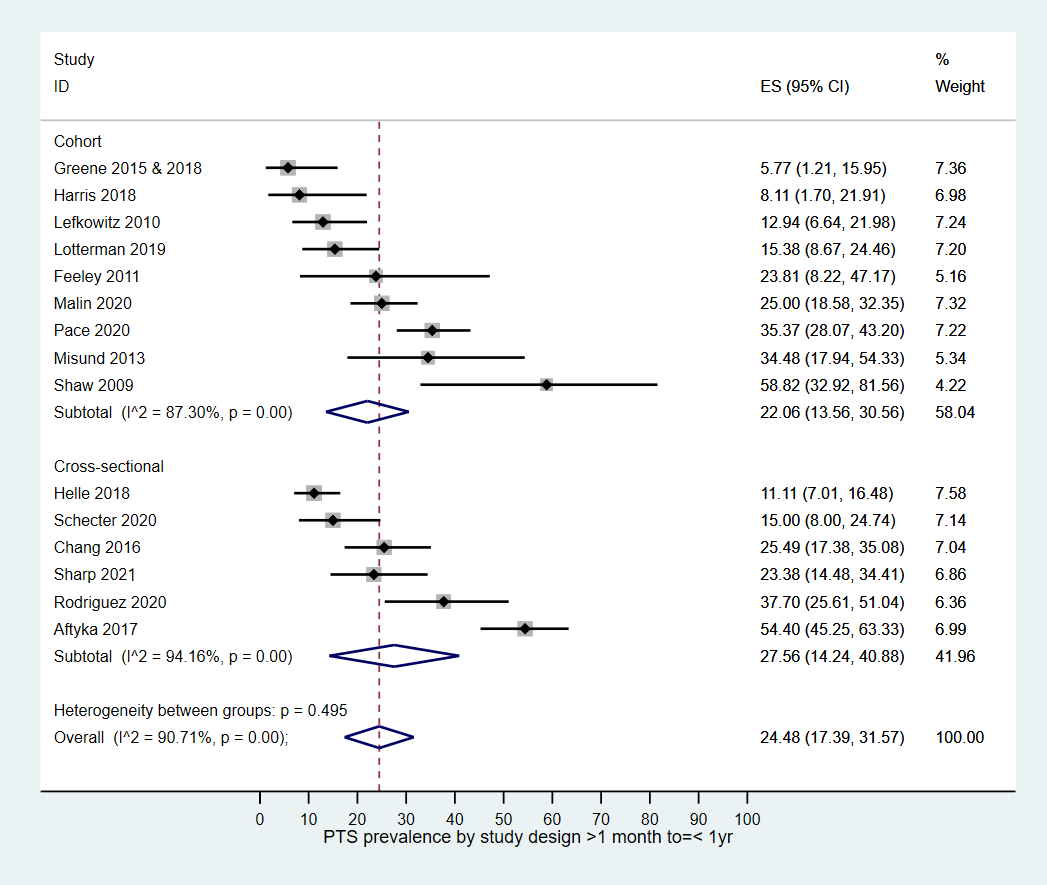


**PTS prevalence > 1 month to ≤ 1 year –selection bias-representativeness***
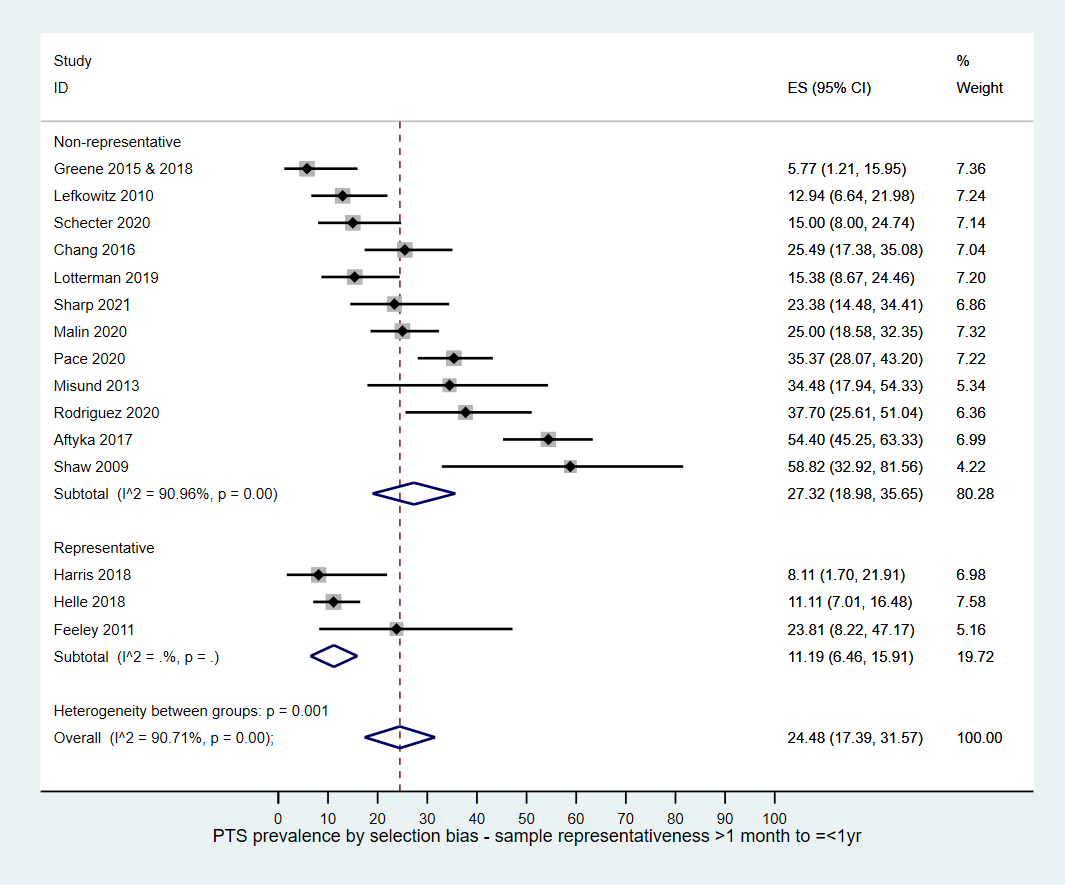


**PTS prevalence > 1 month to ≤ 1 year-PTS symptoms***


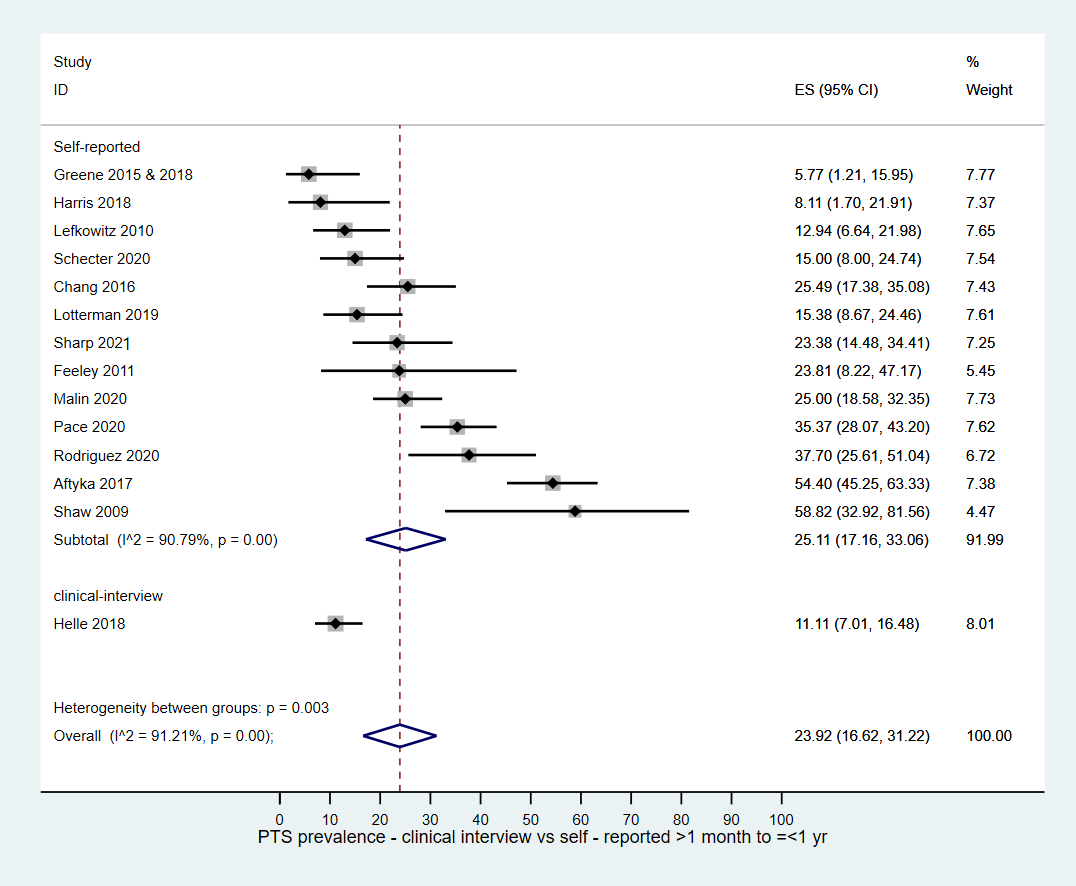


**PTS prevalence > 1 month to ≤ 1 year-parents**


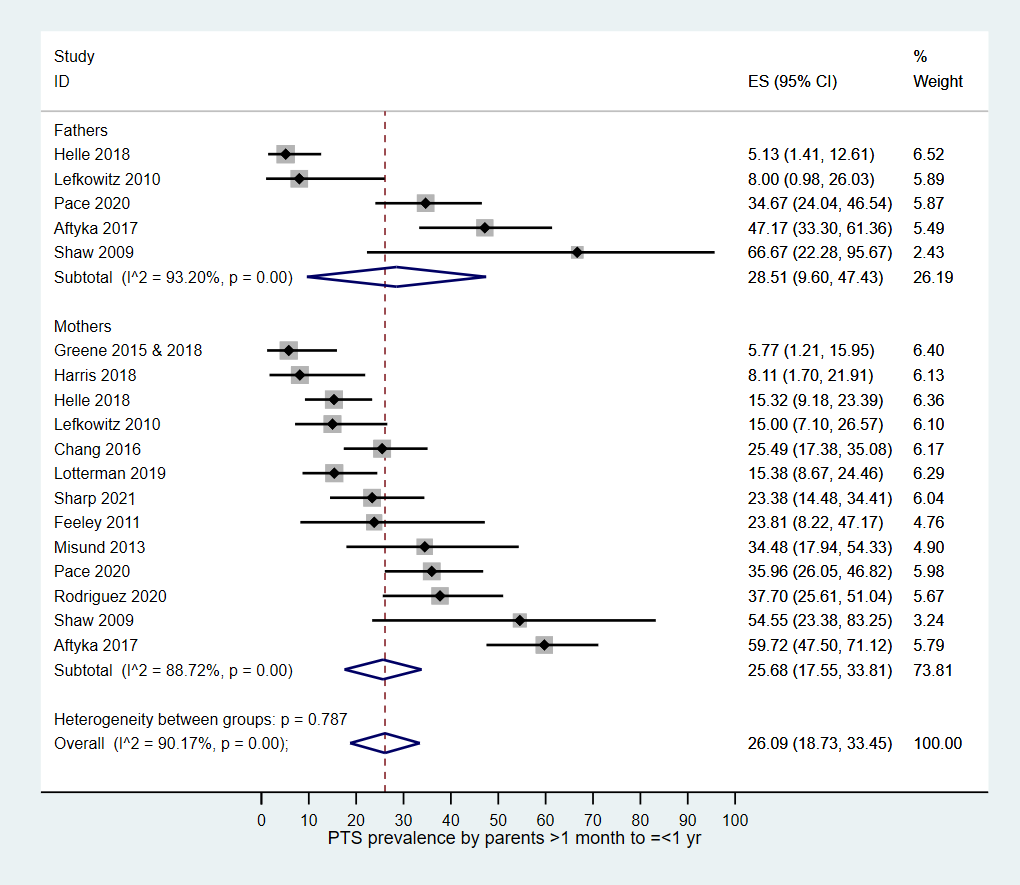


**PTS prevalence > 1 month to ≤ 1 year-prematurity**


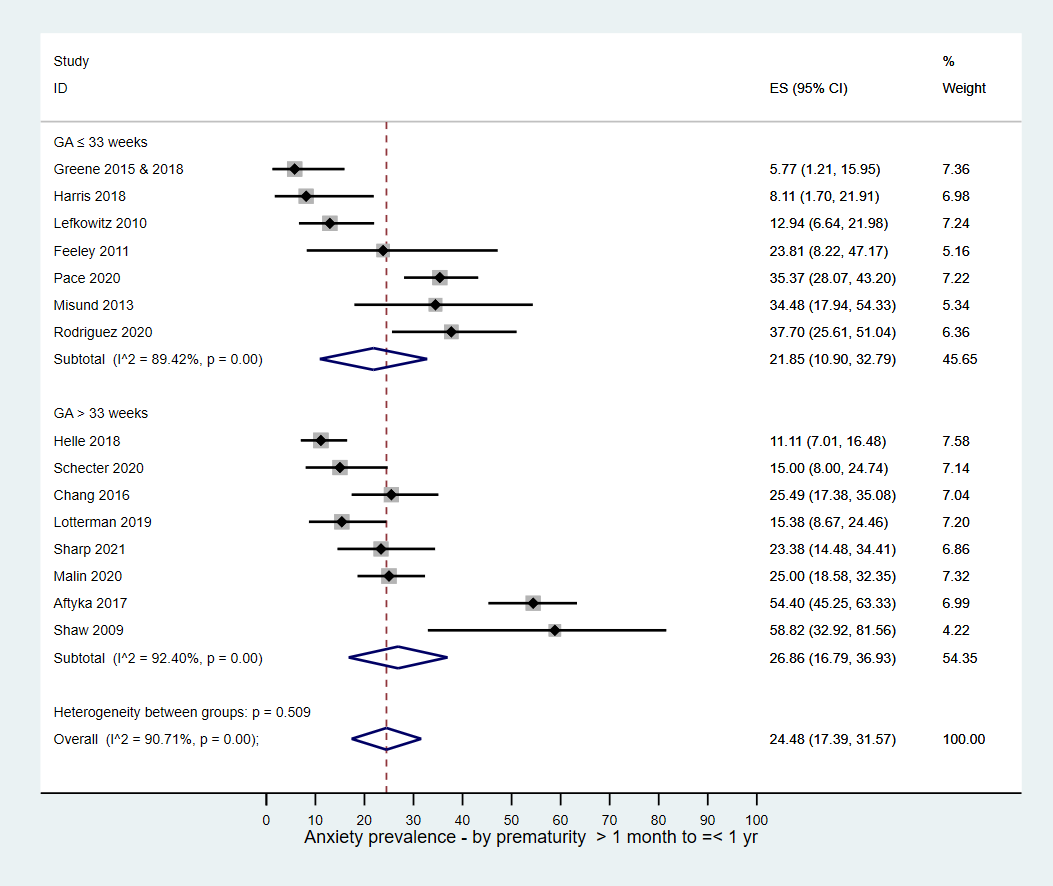


**PTS prevalence > 1 month to ≤ 1 year-measuring scales**


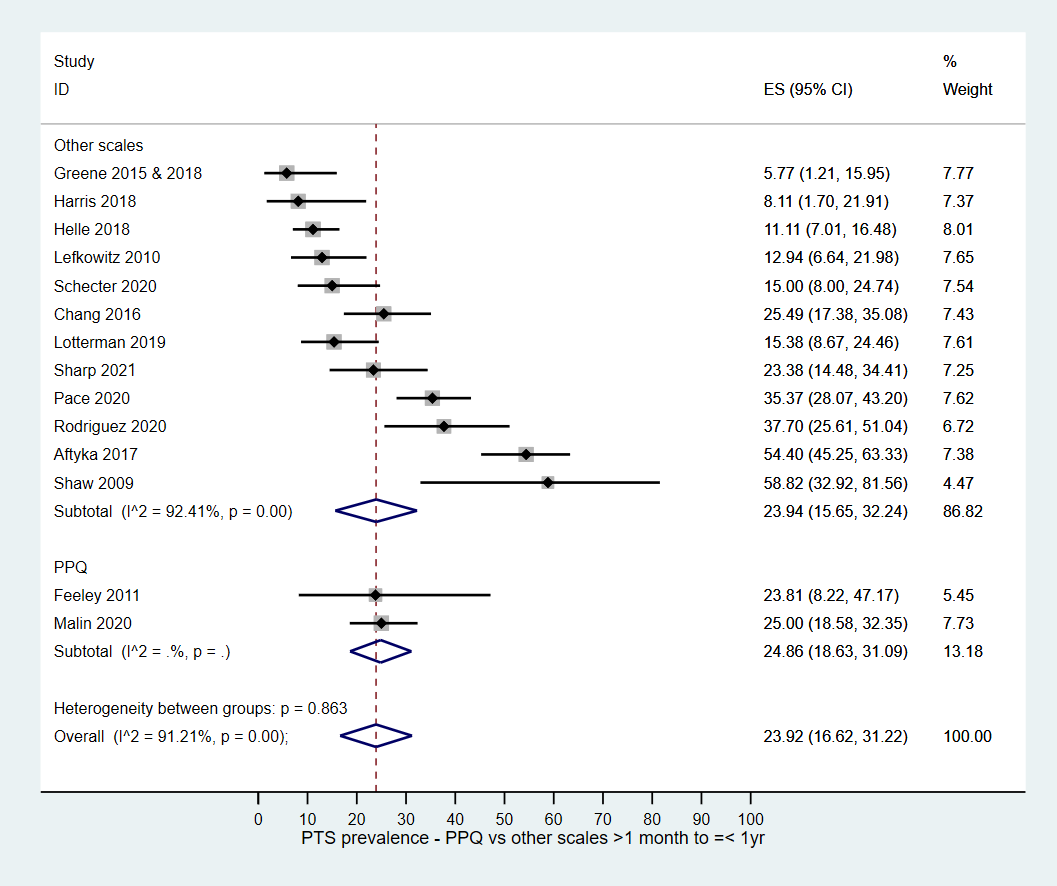


**PTS prevalence >1 yr-study setting***

**
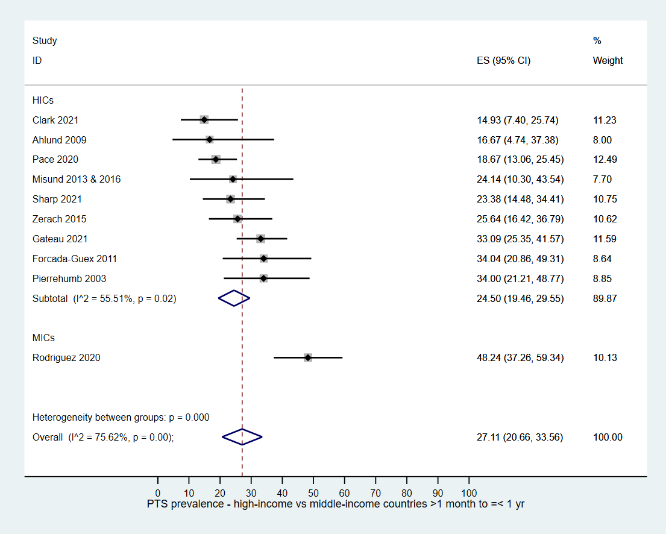
**

**PTS prevalence > 1 year-study design**

**
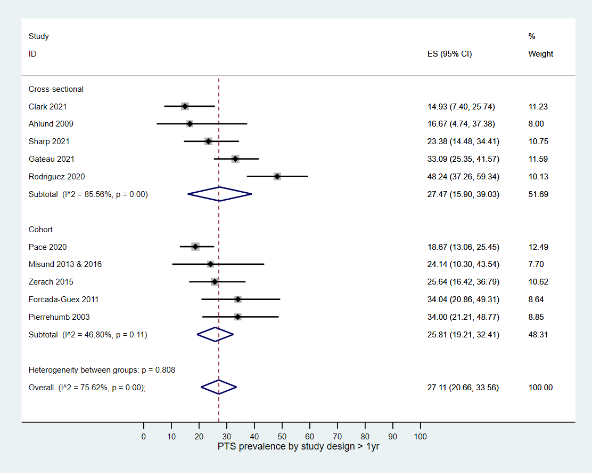
**

**PTS prevalence >1 year-parents***


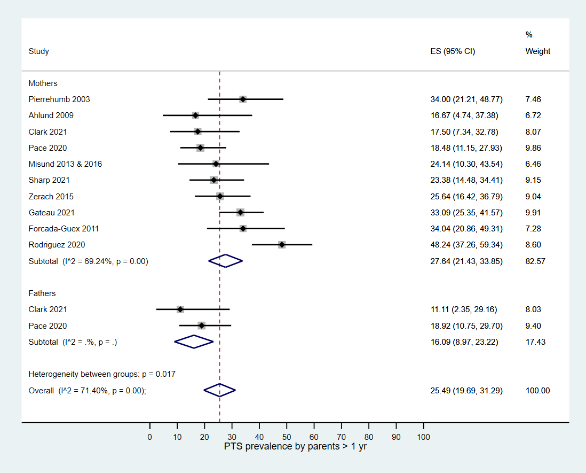


**PTS prevalence >1 year-prematurity**


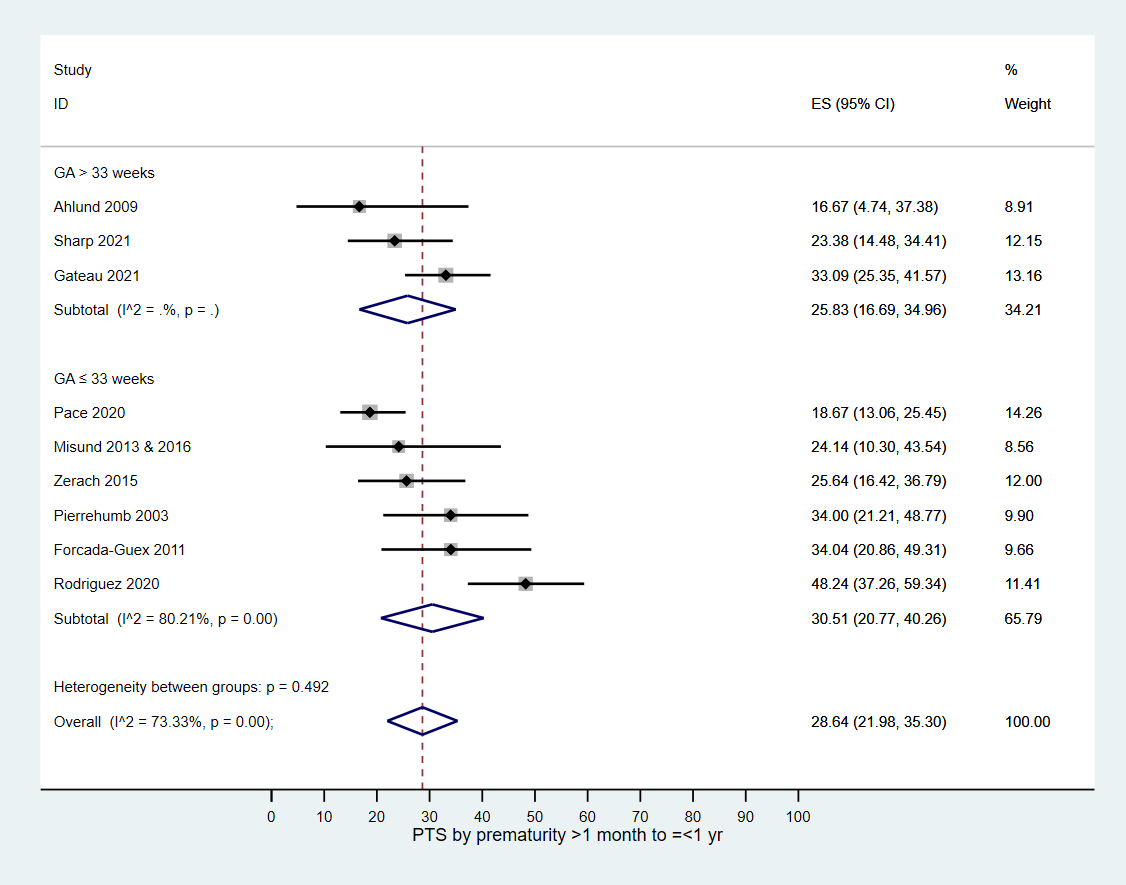


**PTS prevalence > 1 year-measuring scales**


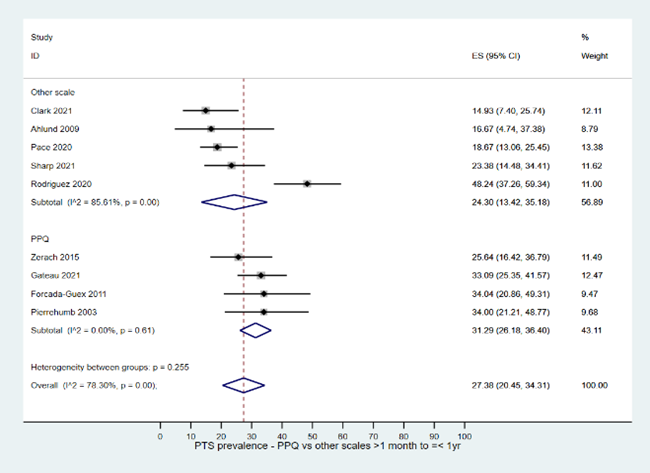

Supplement: Supplementary file 1 [file mmc1.docx]
