# Supplementary material for: Prevalence of anxiety and post-traumatic stress (PTS) among the parents of babies admitted to neonatal units: A systematic review and meta-analysis
Source: eClinicalMedicine. 2021 Dec 21;43:101233. doi: 10.1016/j.eclinm.2021.101233 (PMC8713115; doi:10.1016/j.eclinm.2021.101233)
Supplement: Supplementary file 2 [file mmc2.docx]

**Supplementary Material Headings**

Appendix A: Database: Medline (Ovid MEDLINE® Epub Ahead of Print, In-Process & Other Non-Indexed Citations, Ovid MEDLINE® Daily and Ovid MEDLINE®) 1946 to 24/January/2020

Appendix B Risk of bias assessment tool, adapted from the Risk of Bias Tool for Prevalence Studies developed by Hoy et al

Appendix C: Risk of Bias of included studies - Anxiety

Appendix D: Anxiety prevalence and meta-analyses at different time points

Appendix E: Risk of Bias of included studies - PTS

Appendix F: Post traumatic stress (PTS) prevalence and meta-analyses at different time points
